# The relevance between hypoxia-dependent spatial transcriptomics and the prognosis and efficacy of immunotherapy in claudin-low breast cancer

**DOI:** 10.3389/fimmu.2022.1042835

**Published:** 2023-01-04

**Authors:** Huizhi Sun, Yanlei Li, Yanhui Zhang, Xiulan Zhao, Xueyi Dong, Yuhong Guo, Jing Mo, Na Che, Xinchao Ban, Fan Li, Xiaoyu Bai, Yue Li, Jihui Hao, Danfang Zhang

**Affiliations:** ^1^ Tianjin Medical University Cancer Institute and Hospital, National Clinical Research Center for Cancer, Key Laboratory of Cancer Prevention and Therapy, Tianjin’s Clinical Research Center for Cancer, Tianjin, China; ^2^ Department of Pathology, Tianjin Medical University, Tianjin, China

**Keywords:** breast cancer, hypoxia, spatial transcriptomics, Claudin-low tumor, immune cell infiltration

## Abstract

**Introduction:**

Hypoxia is an important characteristic of solid tumors. However, spatial transcriptomics (ST) of hypoxia-associated heterogeneity is not clear.

**Methods:**

This study integrated Spatial Transcriptomics (ST) with immunofluorescence to demonstrate their spatial distribution in human claudin-low breast cancer MDA-MB-231 engraft. ST spots were clustered with differentially expression genes. The data were combined with hypoxia-specific marker and angiogenesis marker-labeled serial sections to indicate the spatial distribution of hypoxia and hypoxia-inducted transcriptional profile. Moreover, marker genes, cluster-specific hypoxia genes, and their co-essential relationship were identified and mapped in every clusters. The clinicopathological association of marker genes of hypoxia-dependent spatial clusters was explored in 1904 breast cancers from METABRIC database.

**Results:**

The tumor from center to periphery were enriched into five hypoxia-dependent subgroups with differentially expressed genes, which were matched to necrosis, necrosis periphery, hypoxic tumor, adaptive survival tumor, and invasive tumor, respectively. Different subgroups demonstrated distinct hypoxia condition and spatial heterogeneity in biological behavior and signaling pathways. Cox regression analysis showed that the invasive tumor (cluster 0) and hypoxic tumor (cluster 6) score could be served as independent prognostic factors in claudin-low patients. KM analysis indicated that high invasive tumor (cluster 0) and hypoxic tumor (cluster 6) score was associated with poor prognoses of claudin-low patients. Further analysis showed that hypoxia-induced immune checkpoints, such as CD276 and NRP1, upregulation in invasive tumor to block infiltration and activation of B cells and CD8+ T cells to change tumor immune microenvironment.

**Discussion:**

This study reveals hypoxia-dependent spatial heterogeneity in claudin-low breast cancer and highlights its potential value as a predictive biomarker of clinical outcomes and immunotherapy response. The molecules found in this study also provided potential molecular mechanisms and therapeutic targets for subsequent studies.

## Introduction

Breast cancer is a heterogeneous group of neoplasms originating from the mammary duct and acinus system (1). It has been classified into six biologically different subtypes based on gene expression signature, namely, luminal A, luminal B, HER2-enriched, basal-like, claudin-low, and normal-like subtypes (2, 3). Compared to the other subtypes, the claudin-low subtype is characterized by an aggressive histology behavior (4, 5). Claudin-low breast cancers display a low expression of genes with tight junctions and epithelial cell–cell adhesion, including claudins 3, 4, and 7, occludin, and E-cadherin. Epithelial-to-mesenchymal transition (EMT) and cancer stem cell-associated genes were highly expressed in this subtype. It always indicates a triple-negative feature, negative for estrogen receptor (ER), progesterone receptor (PR), and Her2 receptor (6, 7). Claudin-low breast cancer has a poor response to chemotherapy and lacks the targeted therapy (4, 7). Therefore, a better cognition molecular mechanism of claudin-low breast cancer and identifying the protentional treatment targets are urgently needed to improve the prognosis of claudin-low breast cancer.

Another characteristic of claudin-low breast cancer is the high enrichment of immune cell infiltration, IFNγ activation, and high levels of genomic instability (7, 8). Hence, immune checkpoint inhibitors have been proven for use in PD-L1+ metastatic triple-negative breast cancer (TNBC) patients as well as neoadjuvant treatment of TNBC. However, the response to PD-L1+ tumors remains varied (9). The variation has been associated with heterogeneity in the immune cell composition of individual tumors (10, 11). Malignant tumor cells and the tumor microenvironment are responsible for the division of tumor cells and immune cell fate (11). Thus, a deep insight into the regulation mechanism of the tumor microenvironment will provide more immune checkpoint treatment targets.

Hypoxia has a crucial influence on the tumor microenvironment to determine the tumor phenotype of many solid tumors (12). It is also responsible for invasion, metastasis, poor clinical outcome, and resistance to therapies (13). Hypoxic condition promotes the expression of gene products involved in angiogenesis, metabolism, invasion, and metastasis in claudin-low breast cancer (14). Thus, the identification of hypoxia-regulated tumor microenvironment is important both for understanding cancer evolution and for the development of novel therapies (15–17). Traditional transcriptomics using gene chip and RNA-sequencing demonstrate an average transcriptome and lack spatial information. The distribution of hypoxia in solid tumors is unbalanced (18, 19). The tumor with abundant blood supply has mild hypoxia, while the tumor with poor blood supply suffers from severe hypoxia (20). The center of the tumor was hypoxic, while the periphery of the tumor was normoxic. The spatial context of gene transcriptome is important to understand tumor hypoxia and hypoxia-induced gene expressions. Ståhly et al. introduced the spatial transcriptomics (ST) method, which can be used to record the mRNA expression in the spatial context of intact tissue (21–23). Integration of traditional immunofluorescent staining for hypoxia markers and high-dimensional spatial data from different hypoxic microenvironment tissue should therefore facilitate the dissection of hypoxia-induced heterogeneity.

In this study, we established a human claudin-low breast cancer MDA-MB-231 engraft and used an ST technique to investigate the ST feature of claudin-low breast cancer. The ST spots were clustered into five tumor-associated subgroups with differentially expressed genes. These clusters show hypoxia and position-dependent characteristics and unique gene signatures involving encoding proteins with central roles in glucose metabolism, angiogenesis, cell proliferation, programmed cell death, and immune cell infiltration (24, 25). The association of marker genes of hypoxia-dependent spatial clusters with patient survival and tumor progression was explored for 1,904 breast cancers from the METABRIC database. Subsequently, we investigated the correlation between hypoxia-dependent spatial clusters and immune signatures and its ability to predict the efficiency for tumor immune therapy. They will be resources for further investigation of claudin-low breast cancer for prognosis and immune treatment.

## Materials and methods

### Cells and reagents

Human breast cancer MDA-MB-23 cells were cultured in RPMI-1640 medium with 10% FBS, 4 mM L-glutamine, and 1% penicillin–streptomycin. Matrigel (BD Bioscience) was diluted with RPMI-1640 medium in cell transplantation. The Hypoxyprobe-1 Kit (HP1-1000Kit) was purchased from HPI Hypoxyprobe, Inc. (Burlington, USA). Primary antibodies used in this study are listed in **Supplementary Table 1**. Secondary antibodies were purchased from Zhongshan Golden Bridge Biotechnology Co., Ltd. (Beijing, China).

### Tumor-bearing SCID mouse models

The animal experiments were approved by the Tianjin Medical University Contribution Committee (No. 8177110413). All operations were carefully administered to protect the welfare of the animals and prevent them from suffering. Four, 6-week-old female, SCID mice were purchased from Beijing HFK Bioscience Company. Approximately, 1–2×10^6^ MDA-MB-231 cells were subcutaneously injected in the back of the mice (*N* = 4). Tumors were measured every day, and tumor volume was determined using a standard formula (length × width^2^ × 0.52). All mice were sacrificed when average tumor volume reached 0.5 cm^3^. Pimonidazole HCl was injected i.p. (60 mg/kg) 30 min before the animals were sacrificed. The tumors were collected.

### Immunohistochemical staining

Paraffin-embedded sections were stained immunohistochemically. The method is provided in supplementary materials and methods.

### Immunofluorescent staining

Serial frozen sections were used for immunofluorescent staining. The method is provided in supplementary materials and methods.

### Spatial transcriptomics

This procedure includes slice preparation, slide preparation, fixation, staining and imaging, tissue permeabilization, reverse transcription, spatial library preparation and sequencing, library preparation and RNA sequencing, and analysis. The details are provided in supplementary materials and methods.

### Identification of cluster-specific marker genes

Hypoxia genes from the literature are listed in **Supplementary Table 2**. Gene sets with false discovery rate (FDR) adjusted *p*-values below 0.05 were considered significantly enriched in the related clusters. Kyoto Encyclopedia of Genes and Genomes (KEGG) and Gene ontology (GO) slim were used to analyze the involved signaling pathway information. The cluster-specific hypoxia genes were analyzed (ftp.broadinstitute.org://pub/gsea/gene_sets/h.all.v7.1.symbols.gmt).

Marker genes were identified based on the comprehensive analysis of database and gene rank of the LOGFC value in differentially expressed genes of each cluster. The top 100 differentially expressed genes and cluster-specific hypoxia genes of each cluster were imported to the search tool for the retrieval of interacting genes/proteins (STRING) website. The human protein interaction network generated by the STRING database was input into the software Cytoscape 3.8.0. The MCODE was performed to score and enrich the dense region group of the protein interaction network. The BOTTLENECK algorithm of the cytoHubba app was used to screen the top 20 molecules in the protein interaction network. Ten genes with a higher rank, MCODE score, and cytoHubba score were identified as marker genes.

### Clinical significance of cluster-specific marker genes in human Claudin-low breast cancer

The clinical relevance of the cluster-specific marker genes of the MAD-MB-231 engraft was evaluated in the METABRIC database (26, 27). The method is provided in supplementary materials and methods.

## Results

### The spatial transcriptomics of human MDA-MB-231 tumor engrafts indicates hypoxia-dependent spatial heterogeneity

**Figure 1:**
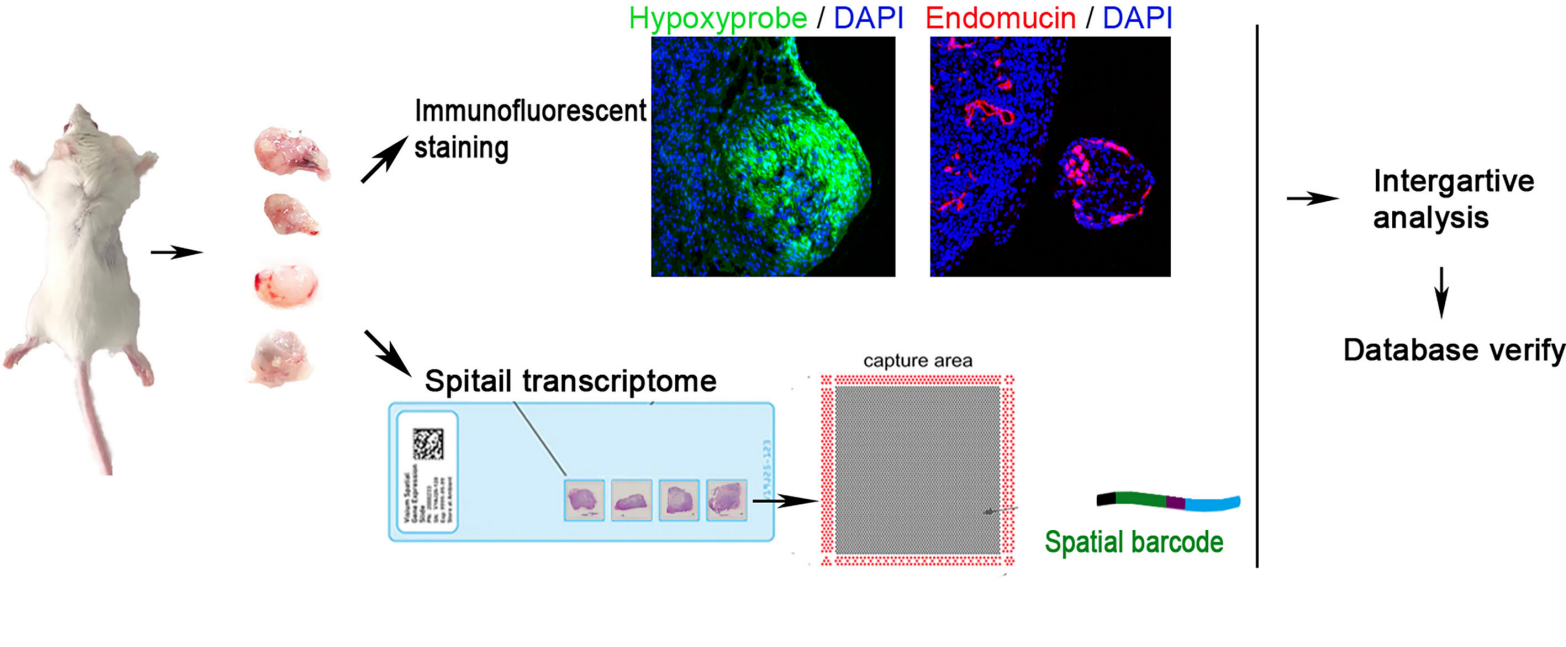
Workflow of claudin-low breast cancer sample processing for ST. Four tumors were collected in this study. They were sectioned and placed on four capture areas of the same slide, respectively.

To reveal the spatial composition of MDA-MB-231 tumor cells at the hypoxic level, we performed ST on four sections from MDA-MB-231 tumor engrafts. **Figure 1** shows the workflow of tumor engraft processing for ST. Transcriptomes from 10,570 spots across four sections were collected. The group-specific alignment software STAR matched the Read2 to the reference genome, human Gsh38, and mouse mm10, and the sequence with the unique alignment position was selected for subsequent analysis. **Figures S1A–D** show the features of ST in this study.

**Figure 2:**
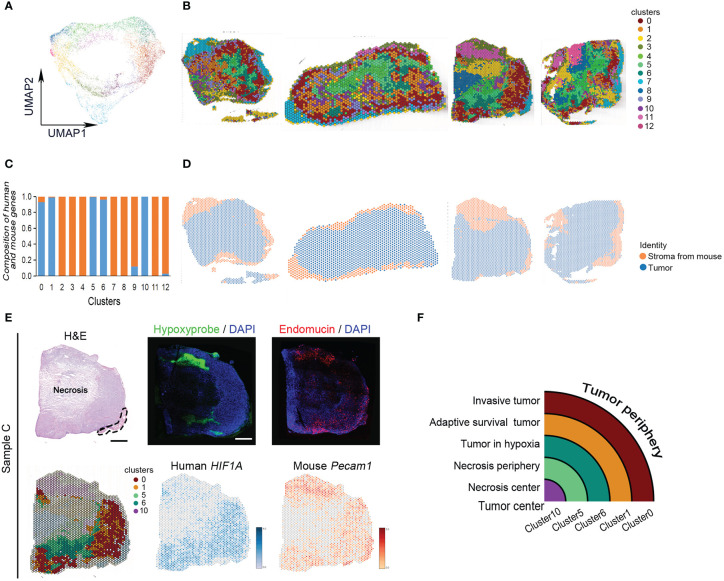
Classification of MDA-MB-231 engrafts. **(A)** UMAP clustering graph for four samples. **(B)** Spatial distribution of all clusters in four samples. **(C)** Composition of human- and mouse-derived differentially expressed genes. **(D)** Spatial distribution of human- and mouse-derived spots in four samples. **(E)** Hematoxylin and eosin **(H, E)** staining of tissue sections and clustering of ST spots of sample **(C)** The dashed line indicates skeletal muscle. Scale bar, 1 mm. Immunofluorescence staining for hypoxyprobe and endomucin of sample **(C)** Spatial feature plots of human HIF1A and mouse Pecam1 expression in tissue sections. **(F)** Ideograph of comparison of hypoxia-dependent ST cluster and tumor tissue region division.

All spots of four tumor samples were clustered into 13 subgroups using uniform manifold approximation and projection (UMAP) (**Figures 2A, B**). There were human tumor-associated clusters and mouse stroma-associated clusters (**Figures 2C, D**). The majority of differentially expressed genes in human tumor-associated clusters 0, 1, 5, 6, and 10 were derived from human genome (**Supplementary Table 3**). The other clusters expressed more mouse skeletal muscle and stroma-specific genes (**Supplementary Table 3**). Every subgroup had its unique differentially expressed genes (**Figures S2A, B**).

The tumors present hypoxia-dependent heterogeneity. From tumor periphery to tumor center, they were divided into cluster 0, cluster 1, cluster 6, cluster 5, and cluster 10 in order. The architecture of these ST clusters was compared with the distribution of human *HIF-1α* and mouse *Pecam*, and the distribution of hypoxia probe and endomucin in serial sections and histological image (**Figure 2E**). The results showed that cluster 0 matched with invasive tumor, cluster 1 matched with adaptive survival tumor, cluster 6 matched with hypoxic tumor, cluster 5 matched with necrosis periphery, and cluster 10 matched with necrosis, (**Figure 2F**).

### Spatial transcriptomics identifies spatial distribution of hypoxia-related genes based on the different hypoxic conditions in MDA-MB-231 tumor engrafts

**Figure 3:**
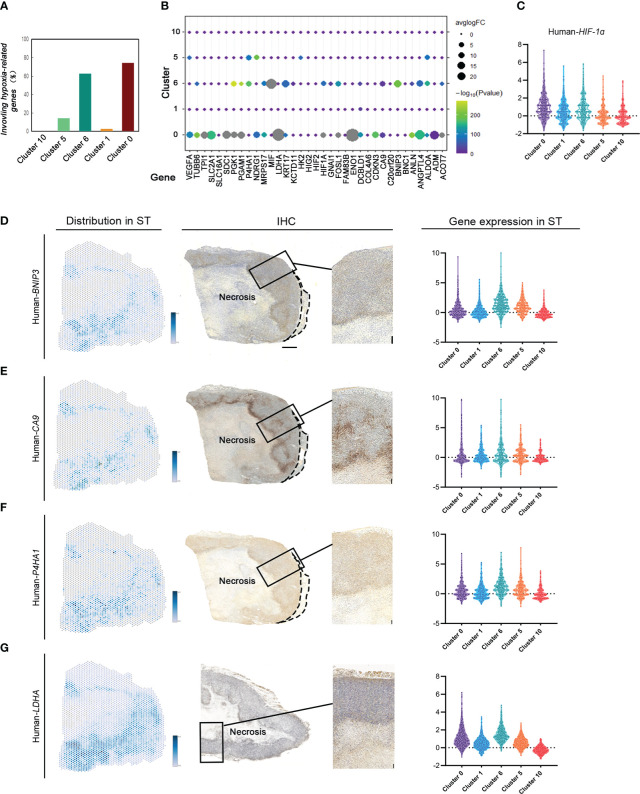
ST identifies hypoxia-related gene expression and distribution based on different hypoxic conditions in MDA-MB-231 engrafts. **(A)** Number of hypoxia-related genes in cluster 0, cluster 1, cluster 6, cluster 5, and cluster 10. **(B)** Distribution of hypoxia-related genes in different clusters. **(C)** Violin plots of HIF1A expression in cluster 0, cluster 1, cluster 6, cluster 5, and cluster 10. **(D–G)** Spatial feature plots of Human BINP3, CA9, P4HA1, and LDHA in sample **(C)** IHC for human BINP3, CA9, and P4HA1 in sample C and LDHA in sample B (scale bar, 1 mm). Violin plots of these genes’ expression in cluster 0, cluster 1, cluster 6, cluster 5, and cluster 10.

To investigate the gene signature regulating tumor response to various hypoxic conditions in different areas, 35 key hypoxia-related genes reported by literature were detected to indicate their expression and spatial distribution in the same tumor. There were 28 genes differentially expressed in MDA-MB-231 tumors. Interestingly, these hypoxia-associated genes were differentially expressed and distributed in these groups (**Figures 3A, B**). Most (74.29%) hypoxia-related genes were differentially expressed in cluster 0, the invasive tumor. Approximately over 62.86% of them were differentially expressed in cluster 6, the hypoxic tumor. Hypoxia-related genes were rarely expressed in other subgroups (**Figures 3A–C**).

The expression of tumor area-specific hypoxia genes was validated with IHC. The expression of *BINP3*, inducing apoptosis, in cluster 6 correlated with most of them, while there was no significant regulation between *BINP3* and the other genes in other groups. The results in both ST and IHC identified that *BINP3* expression in cluster 6 was significantly different from that in others (**Figure 3D**). *CA9* expression in cluster 0 and cluster 6 was significantly different from that in others (**Figure 3E**). *P4HA1* was differentially expressed in cluster 0 compared to the other subgroups **(Figure 3F**). *LDHA* had a high level of expression in the claudin-low breast cancer tissue. There was no significant difference in *LDHA* expression under different hypoxic conditions (**Figure 3G**).

### The marker genes and functions of hypoxia-dependent spatial clusters

**Figure 4:**
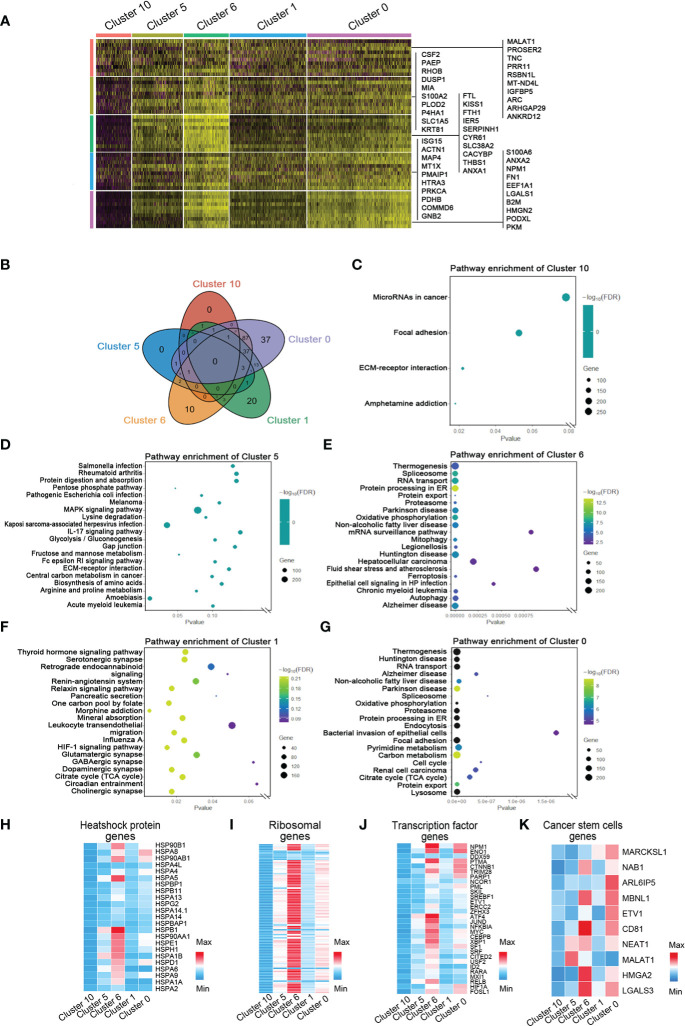
Marker genes and the relative signaling pathways of five hypoxia-dependent spatial clusters. **(A)** Heatmap of expression of maker genes in hypoxia-dependent spatial clusters. **(B)** The Venn diagram of the gene ontology (GO) pathway in cluster 0, cluster 1, cluster 6, cluster 5, and cluster 10. **(C)** Dot plot of the top 4 GO terms of differentially expressed genes in cluster 10. **(D)** Dot plot of the top 20 GO terms of differentially expressed genes in cluster 5. **(E)** Dot plot of the top 20 GO terms of differentially expressed genes in cluster 6. **(F)** Dot plot of the top 20 GO terms of differentially expressed genes in cluster 1. **(G)** Dot plot of the top 20 GO terms of differentially expressed genes in cluster 0. **(H–K)** Expression-level scaled heatmap of heat shock proteins, genes, ribosomal genes, transcriptional factor genes, and cancer stem cell genes of cluster 0, cluster 1, cluster 6, cluster 5, and cluster 10.

To investigate the marker genes in invasive tumor, adaptive survival tumor, hypoxic tumor, necrosis periphery, and necrosis, we used correlation analysis, string map, MCODE, and cytoHubba in the software Cytoscape to identify key genes in the network of the top 100 genes and the cluster-specific hypoxia genes in every cluster. Ten marker genes in each cluster were sifted (**Figure 4A** and **Figures S3**, **S7**). The hypoxia-dependent spatial clusters showed different gene signatures involving encoding proteins with central roles in glucose metabolism, angiogenesis, cell proliferation, programmed cell death, and extracellular matrix synthesis.

To detect the function of the transcriptome in each region, GO slim and KEGG signaling pathway analysis were used to analyze the top 100 genes in every cluster. The most differentially expressed genes in invasive tumor (cluster 0) are involved in the metabolism-related signaling pathway, such as thermogenesis, oxidative phosphorylation, pyrimidine metabolism, carbon metabolism, and citrate cycle (TCA cycle) (**Figures 4B–G** and **Figure S8**). Hypoxic tumor (cluster 6) was associated with several programmed cell death signaling pathways, involving autophagy and ferroptosis. The function of these genes was consistent with their location and behavior in tumor. The heatmaps showed the distribution of heat shock protein family members, ribosomal genes, and transcriptional regulator expression in each group (**Figures 4H–K**). A variety of heat shock proteins and ribosomal genes were highly expressed in the hypoxic tumor (cluster 6). **Figure 4J** shows that each tumor region has its own transcriptional factor expression profile. *NPM1*, *ENO1*, and *CTNNB1* are highly expressed in the invasive tumor, and *ATF4*, *JUND*, and *MYC* are highly expressed in the hypoxia group (**Figure 4J**). Surprisingly, *HIF1A* expression in the invasive tumor was higher than that in the hypoxic tumor (**Figure 4J**). The gene expression profiles generally also reflected the functional requirements of tumor cells’ response to hypoxic microenvironment.

### The marker genes of hypoxia-dependent spatial clusters in MDA-MB-231 tumor engrafts differentially expressed between different human breast cancer subtypes

**Figure 5:**
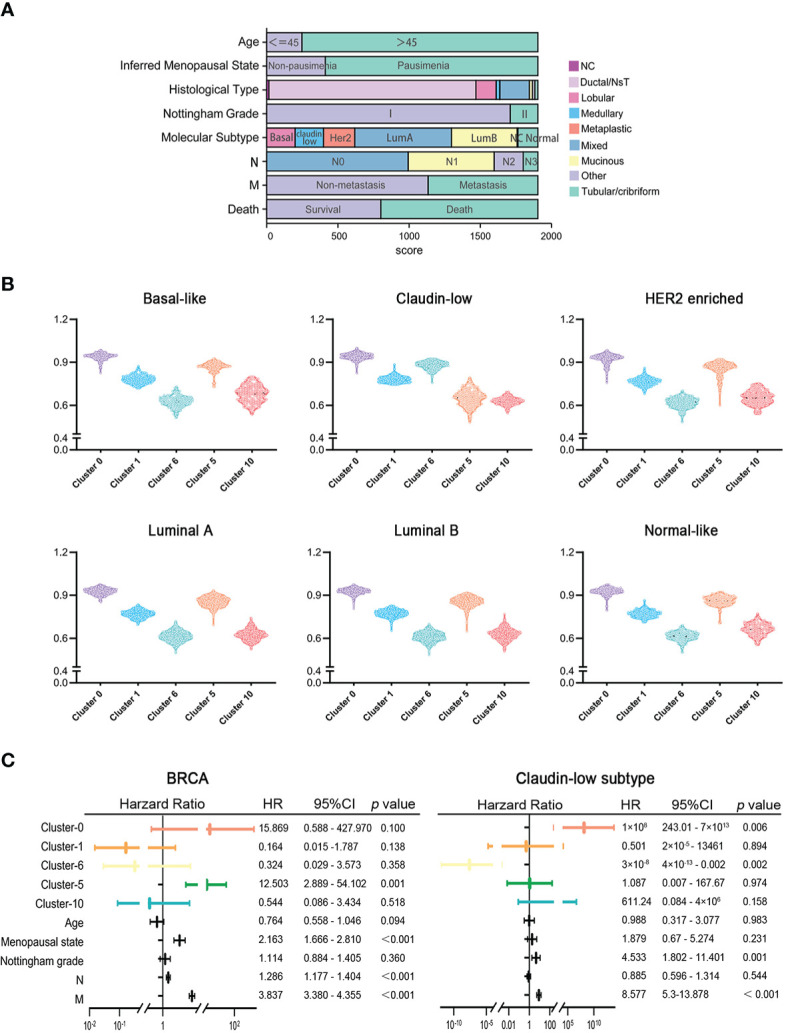
**(A)** The clinicopathological factors of 1,904 human breast cancers. **(B)** Comparisons of hypoxia-dependent spatial subgroup scores of cluster 0, cluster 1, cluster 6, cluster 5, and cluster 10 in different breast cancer subtypes. **(C)** Multivariate Cox proportional hazards regression model for hypoxia-dependent spatial clusters score and clinicopathological factors in all breast cancer and claudin-low subtypes.

To explore the clinical significance of marker genes of hypoxia-dependent spatial clusters in MDA-MB-231 tumor engrafts, the gene expression data of 1,904 breast cancers from METABRIC were used to perform single-sample gene set enrichment analysis (ssGSEA) to calculate the score for cluster 0, cluster 1, cluster 6, cluster 5, and cluster 10. These samples were classified into six subtypes: normal-like, luminal A, luminal B, HER2-enriched, claudin-low, and basal-like (**Figure 5A**). **Supplementary Table 4** lists the ssGSEA score and clinicopathological factors of each sample. The difference in marker gene score of hypoxia-dependent spatial clusters was compared between the normal-like, luminal A, luminal B, HER2-enriched, claudin-low, and basal-like subtypes. These subtypes had a significant difference in cluster 0, cluster 1, cluster 6, cluster 5, and cluster 10 scores (ANOVA, *p* < 0.0001, **Figure 5B**). The expression pattern of marker genes of hypoxia-dependent spatial clusters in the claudin-low subtype was distinguished from the pattern in the others. Claudin-low subtype showed a higher cluster 6 score when compared to the others. The cluster 5 expression in the claudin-low subtype was lower than that in others (**Figure 5B**). The results suggested that the marker gene score of hypoxia-dependent spatial clusters might have various effects on different human breast cancer subtypes.

### High cluster 0 and high cluster 6 scores were associated with the poor prognosis of the claudin-low subtype of breast cancer

To investigate the influence of hypoxia-dependent spatial cluster genes on different breast cancer subtypes, multivariate Cox regression analysis was performed to analyze whether the expression of hypoxia-dependent spatial cluster genes can be an independent prognostic factor for breast cancer patients in different subtypes. Cluster 0, cluster 1, cluster 6, cluster 5, and cluster 10 scores and other clinicopathological factors served as covariates. The results indicated that cluster 0, cluster 6, Nottingham grade, and distant metastases were significantly associated with the survival of claudin-low subtype patients (**Figure 5C**). Like Nottingham grade and distant metastases, cluster 0 and cluster 6 genes could be independent prognostic factors for the claudin-low subtype (**Figure 5C**). In all breast cancer and basal-like subtypes, cluster 5 genes as well as distant metastases were significantly related to the survival of patients (**Figures 5C**, **S9A**). However, hypoxia-dependent spatial cluster genes had no significant effect on the survival of patients in HER2-enriched, luminal A, luminal B, and normal-like subtype (**Figures S9B–E**).

**Figure 6:**
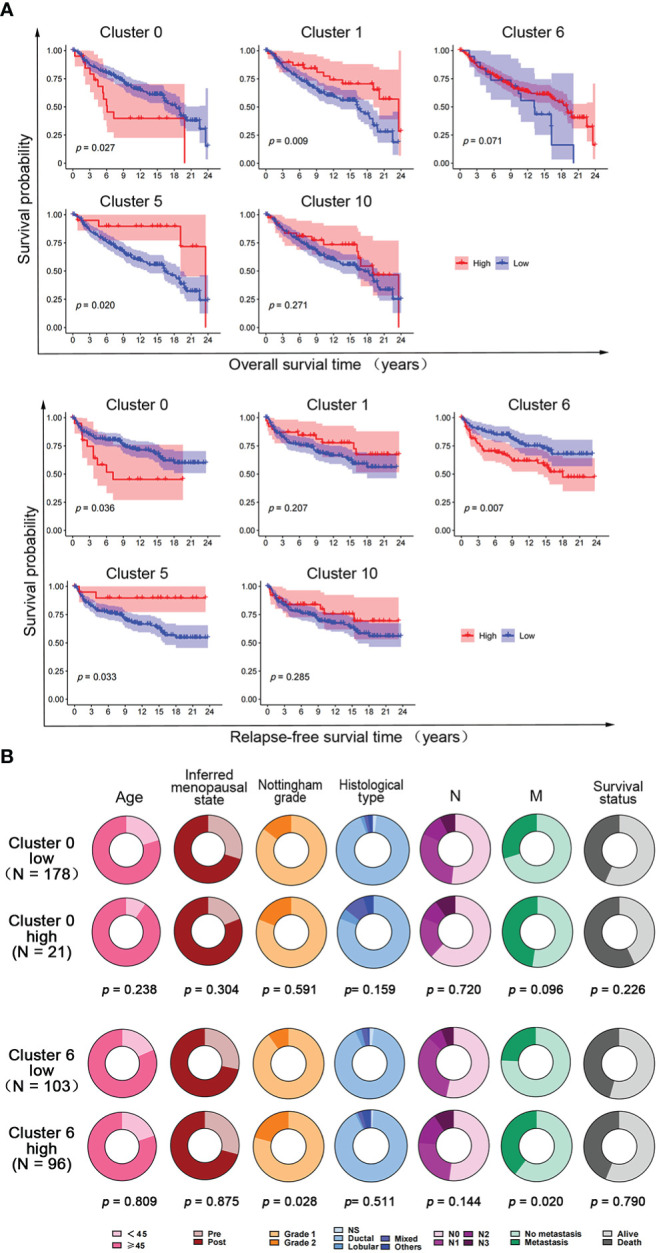
The effect of hypoxia-dependent spatial subgroup scores of cluster 0, cluster 1, cluster 6, cluster 5, and cluster 10 on survival and clinicopathological factors in human claudin-low breast cancer. **(A)** Kaplan–Meier OS and RFS survival plot of hypoxia-dependent spatial cluster score of cluster 0, cluster 1, cluster 6, cluster 5, and cluster 10 in the claudin-low subtype. **(B)** The distribution of clinicopathologic factors for low and high groups of cluster 0 gene score and cluster 6 gene score in human claudin-low breast cancer. Pie charts indicated the chi-squared test results.

To further investigate the correlation between the expression of hypoxia-dependent spatial cluster genes and prognosis of claudin-low subtype, Kaplan–Meier (KM) analysis was performed based on cluster 0, cluster 1, cluster 6, cluster 5, and cluster 10 scores. Patients were dichotomized into high and low groups, respectively, according to the auto select best cutoff score of each cancer. The result of the KM analysis indicated that patients with high cluster 0 gene expression had significantly poorer clinical outcomes of overall survival (OS) and relapse-free survival (RFS) than those with low cluster 0 gene expression in the claudin-low subtype (**Figure 6A**). The RFS of patients with a high cluster 6 score was worse than that with a low cluster 6 score in this subtype (**Figure 6A**). High cluster 1 and cluster 5 gene expression had a negative effect on the OS and RFS of patients in the claudin-low subtype. However, there was no significant difference in OS and RFS between cluster 10 expression groups in the claudin-low subtype (**Figure 6A**). Based on the results of Cox analysis and KM survival analysis, cluster 0 and cluster 6 ssGSEA scores were independent prognostic factors for the claudin-low subtype.

To explore the effect of cluster 0 and cluster 6 genes on clinicopathological factors, the chi-square test was used to compare the difference in clinicopathological factors between the high-score and the low-score groups in the claudin-low subtype. The results showed that the cluster 0 high-score group and the cluster 6 high-score group had more metastasis and poorer survival than the cluster 0 low-score group and the cluster 6 high-score group, respectively (**Figure 6B**). Tumors in the cluster 6 high-score group showed a higher Nottingham grade than those in the cluster 6 low-score group. There was no difference in other clinicopathological factors (**Figure 6B**).

### Cluster 0 and cluster 6 scores were negatively correlated with immune cell infiltration in the claudin-low subtype of human breast cancer

To detect the correlation between breast cancer space-dependent cluster-related genes and tumor immunity, immune cell infiltration was analyzed in different subtypes. Immune cell infiltration in tumors of the claudin-low subtype was significantly different from those in the other subtypes (ANOVA, *p* < 0.0001, **Figure S10**). More immune cells, including B cells, TIL, CD8+ T cells, T helper cells, Th1 cells, Th2 cells, follicular helper T (Tfh) cells, natural killer (NK) cells, dendritic cells (DCs), macrophages, and neutrophils, are infiltrated in the claudin-low subtype than the others. Pearson correlation analysis showed that cluster 0, cluster 6, and cluster 5 scores were negatively correlated with different immune cell infiltrations, such as B cells, TIL, CD8+ T cells, Th1 cells, Th2 cells, Tfh cells, NK cells, and DCs, in the claudin-low subtype of breast cancer (**Figure S11A**). Cluster 0 and cluster 6 scores had a positive effect on macrophage infiltration in the claudin-low subtype of breast cancer (**Figure S11A**). Moreover, cluster 0, cluster 6, and cluster 5 scores had a significant and negative correlation with T cell function and inflammation (**Figure S11B**). Interestingly, cluster 0, cluster 6, and cluster 5 scores inhibited the expression of inflammation-related genes and promoted the expression of para-inflammation-related genes (**Figure S11B**). Cluster 0, cluster 6, and cluster 5 scores were negatively associated with checkpoint gene score. However, the relationship was not significant (**Figure S11B**).

In view of the influence of cluster 0 and cluster 6 gene on the survival of the claudin-low subtype, we compared the differences in immune cell infiltration and immune function between the high-expression group and the low-expression group. There were fewer B cells, tumor-infiltrating lymphocytes (TIL), CD8+ T cells, T helper cell 1 (Th1 cells), T helper cell 2 (Th2 cells), follicular helper T cell (Tfh), natural killer (NK) cells, dendritic cells (DCs), and neutrophils in the cluster 0 high-score group than in the cluster 0 low-score group (**Figure S11C**). Macrophages that infiltrated the cluster 0 high-score tumors were more than those in the cluster 0 low-score tumors (**Figure S11C**). Tumor infiltration of B cells, TIL, T helper cells, Th2 cells, Tfh, Treg, NK cells, macrophages, DCs, and aDCs was decreased in the cluster 6 high-score tumors (**Figure S11D**). Compared with the cluster 0 and cluster 6 low-score group, T-cell stimulation, cytolytic activity, chemokine receptors (CCR), and inflammation were inhibited in the cluster 0 and cluster 6 high-score group (**Figure S11C, D**).

To explore the mechanism of hypoxic tumor microenvironment and regulate the immune activity of claudin-low breast cancer, the expression of checkpoint genes (**Supplementary Table 5**) was indicated in the ST slides. The results showed that *CD276*, *CD40*, *CD274*, *CD44*, and *NRP1* expression was exclusively upregulated in cluster 0 (invasive tumor, **Figure S12A**). *TNFSF9*, *CD276*, *CD40*, *CD274*, and *CD44* expression was downregulated in cluster 6 (hypoxic tumor, **Figure S12A**). This gene expression was validated in human claudin-low breast cancer. CD276 and NPR1 expression increased in the cluster 0 high-score tumors than in the cluster 0 low-score tumors (**Figure S12B**). CD40 expression decreased in the cluster 6 high-score tumors than in the cluster 6 low-score tumors (**Figure S12C**). Moreover, survival analysis demonstrated that CD276 and NPR1 expression had a negative influence on OS and RFS of claudin-low subtype breast cancer (**Figures S12D, E**).

## Discussion

Hypoxia is one of the important microenvironment characteristics of solid tumors, which leads to the enhancement of tumor invasion, metastasis, and drug resistance (28). The distribution of blood vessels in tumor tissue is not balanced (20), and the distance between tumor cells and blood vessels in different positions is different, which leads to the difference in hypoxia level in tumor tissues (29). Thus, uneven distribution of hypoxia is responsible for tumor heterogeneity. In this study, ST technology was used for the first time to show the hypoxia-dependent spatial transcriptome in a human breast cancer engraft. The clinical significance was validated in the breast cancer METABRIC database.

From the center to the edge, an MDA-MB-231 tumor mass was categorized into necrosis, necrosis periphery, hypoxic tumor, adaptive survival tumor, and invasive tumor based on hypoxic state and transcriptome signature. Every area had its own unique expression profile. The necrosis region (cluster 10) in the tumor center expressed only several genes. A few tumor cells survived at the edge of necrosis (cluster 5). They expressed arginine and proline metabolism-related molecules and drug resistance-related genes in high levels. MDA-MB-231 tumor cells matching cluster 6 were positive for hypoxia probe, which showed that this tumor region was the most hypoxic region. There was a high expression of several genes promoting and antagonizing programmed cell death. The tumor cells in cluster 1 adapted to hypoxia and survived, which expressed genes in the HIF-1α pathway and hypermetabolic pathway. Tumor cells at the edge of the tumor (cluster 0) expressed genes relating to invasion, tumor metabolism, and immunosuppression. A tumor is a dynamic interconnected ecosystem. Under the influence of different hypoxic microenvironments, various gene networks are activated to determine the tumor cells’ fate in different regions. They obtained different results, such as death, survival, or invasion and metastasis, so that the whole tumor can survive and even progress in a hypoxic environment.

Hypoxia is not only related to the prognosis of tumor patients but also one of the reasons for the failure of chemotherapy, radiotherapy, and anti-angiogenesis therapy (30). Therefore, hypoxia has become an important target of tumor treatment (29, 31, 32). A number of studies have explored the expression of hypoxia-induced genes in various malignant tumors and identified a number of groups of markers to predict the prognosis of cancer patients and the efficacy of anti-hypoxia therapy (16, 17). In this study, we also detected the spatial transcriptional distribution of 35 hypoxia-related genes and found that the different tumor regions that activated the hypoxia-related gene spectrum have their own characteristics. The expression of *BINP3*, which regulates apoptosis (33), is high in the hypoxic area, and *CA9* is different from that in the surrounding area in the hypoxic and invasion areas. *P4HA1* was expressed in many regions, but there was significant difference in the necrosis periphery. The expression level of *LDHA* was high in the whole tumor tissue, and the difference was most significant in the invasion area. The above results were also confirmed by immunohistochemical staining. The distribution of hypoxia-related genes in tumor tissues is not exactly the same as the corresponding hypoxia status, which is consistent with previous research results (16, 34). In addition to HIF-1α, there are other pathways that can regulate the activation of related pathways (35, 36). These results also indicated that a single marker cannot reflect the tumor hypoxia state, and the combination of multiple markers should be used for comprehensive judgment. Hypoxia-related genes not only communicate with each other, but also play a key role in the gene regulatory network of various regions. Among these factors, *HIF1A*, the most powerful regulatory gene, is the core of this regulatory network. It targeted *ALDO, LDHA, ENO1, PGK1, CA9*, and other genes in the invasive tumor and the hypoxic tumor. Moreover, hypoxia-inducible factor 1-alpha (HIF-1α) also activated the transcription of other differentiated expression genes. HIF-1α binds to two HREs 441 and 423 base pairs upstream of the transcriptional start site of *LGALS1* (37, 38)*. NPM1* is also a transcription factor that can regulate the expression of PD-L1 (39). *APE1* and its interactor, *NPM1*, protect cancer cells from cytotoxicity from platinum compounds in claudin-low breast cancer (40). Therefore, the corresponding genes can also be used as predictors of hypoxia and therapeutic targets for the anti-hypoxia microenvironment.

In the present study, we investigated the clinical significance of the hypoxia-dependent spatial transcriptome of the MDA-MB-231 engraft. Marker genes of the hypoxia-dependent spatial transcriptome were verified, and the ssGSEA scores of marker genes of five clusters were validated in the breast cancer METABRIC database. The marker gene expression patterns of invasive tumor (cluster 0), hypoxic tumor (cluster 6), and necrosis periphery (cluster 5) in the claudin-low subtype were distinguished from the other breast cancer subtypes. Cox analysis indicated that invasive tumor gene score and hypoxic gene score were independent predictive factors for OS of claudin-low breast cancer. The high expression of invasive tumor gene score (cluster 0) was associated with poor clinical outcome in the claudin-low breast cancer subtype. These results demonstrated that an invasive tumor gene cluster is helpful for predicting claudin-low subtype prognosis.

Considering the impact of cluster 0 genes on the survival of this subtype of breast cancer, we further explored the relationship between cluster 0 genes and other clinicopathological indicators. The high expression of cluster 0 genes was correlated with recurrence and metastasis, but not with tumor differentiation and lymph node metastasis. These results suggest that cluster 0 genes regulated tumor metastasis through other factors in the claudin-low subtype. The immune microenvironment is critical for tumor development and progression. Cancer stem cells (CSCs) can escape immune surveillance through various methods (41, 42). Moreover, head and neck squamous cell carcinoma cells, most likely CSCs, frequently metastasize to survive in cervical lymph nodes, which are enriched with immune cells (43). It has been reported that transforming growth factor-b induced CSCs of CD80 expression, an immune cell surface ligand, to block cytotoxic T-cell activity and mediate tumor resistance to adoptive cytotoxic T-cell transfer-based immunotherapy (41). This study found that in claudin-low breast cancers, cluster 0 genes were negatively correlated with the infiltration of most immune cells and the scores of T-cell immune activation and cytotoxicity. In cluster 0 genes, high-expression tumors, B cells, TIL, CD8, and other cells decreased. In the tumor margin zone with the strongest tumor invasion and metastasis ability, the genes related to CSCs of this subtype are also highly expressed. The transcriptome of the hypoxia-induced invasive tumor (cluster 0) inhibited the infiltration of immune cells. The resulting tumor immunosuppression enhances the survival ability of CSCs and promotes tumor invasion and metastasis.

Human claudin-low breast cancer is enriched with immune cells. There were more immune cell infiltrates than other subtypes of breast cancer. In this cohort, it was also confirmed that the infiltration of B cells, T cells, NK cells, macrophages, and neutrophils in the claudin-low subtype was higher than that in other subtypes. However, clinical studies showed that although there are sufficient lymphocytes in this subtype, many patients are still insensitive to immune checkpoint therapy. The high expression of cluster 0-related genes found in this study may be related to this. The highly expressed genes in cluster 0 included a lot of immune checkpoint genes, including CD276 and Neuropilin-1 (NPR1), in addition to CD274. CD276 expression was responsible for the poor response to anti-PD-1 immunotherapy in non-small cell lung cancer and ovarian cancer. CD276-expressing tumors were associated with the exclusion of CD8+ tumor-infiltrating lymphocytes. CD276 also suppressed the activity of the stress-activated transcription factor Nrf2 to induce HIF-1α stabilization (44). In the present study, cluster 0 genes in the claudin-low subtype are inversely correlated with infiltrated CD8+ T cells. NPR1 is a marker for thymically derived murine regulatory T cells (Tregs). It is crucial for their suppression of anti-tumor immunity (45). Chang Liu et al. reported that an NRP1 deletion of CD8+ T cell substantially protected patients from tumor re-challenge and promoted response to anti-PD1 immunotherapy in head and neck squamous cell carcinoma (42, 46). NRP1 expression was correlated with Bcl2 loss, leading to antigen-dependent maintenance of CD8+ T-cell exhaustion and impaired memory differentiation (46). These results suggest that CD276 in invasive tumor (cluster 0) blocks the infiltration of CD8+ T cells to change the tumor immune microenvironment. Moreover, NRP1 overexpression accelerated CD8+ T-cell exhaustion in invasive tumor (cluster 0). The lack of insufficient infiltration of activated CD8+ T cells into the tumor microenvironment leads to unresponsiveness to immunotherapy (**Figure S13**). Therefore, after inhibition of CD274, other immune checkpoint genes still exert immunosuppressive effects, resulting in the limited efficacy of the PD-L1 inhibitor. These highlights the interesting role of CD276 and NRP1 as therapeutic targets of a claudin-low subtype tumor unresponsive to the PD-L1 inhibitor.

This study first reveals hypoxic and spatial heterogeneity in claudin-low breast cancer and highlights some communication network controlling cell fate and the tumor immune microenvironment under different hypoxic conditions. The hypoxia-dependent tumor segmentation and marker gene ssGSEA analysis can not only predict prognosis and immunotherapeutic effect, but also provide more potential candidates for molecular mechanism and tumor therapeutic targets for future research.

## Data availability statement

The datasets presented in this study can be found in online repositories. The names of the repository/repositories and accession number(s) can be found below: 10.6084/m9.figshare.21695735.v1.

## Ethics statement

The animal experiments were approved by the Tianjin Medical University Contribution Committee. Written informed consent was obtained from the individual(s) for the publication of any potentially identifiable images or data included in this article.

## Author contributions

Conceptualization: DZ and JH. Methodology: XZ and DZ. Investigation: HS, YLL, YZ, XD, YG, JM, NC and XCB. Formal Analysis: HS, FL, XYB and YL. Writing—Original Draft: HS. Writing—Review and Editing: DZ. Funding Acquisition: XZ, JH and DZ. Supervision: JH and DZ.

## Supplementary material

Supplementary Figure 1 A Spatial Transcriptomic Atlas of human claudin-low MDA-MB-231 engrafts. **(A)** Distribution of all expression gene numbers in four samples, distribution of all counts in four samples, distribution of mitochondrial gene in four samples, distribution of hemoglobin gene expression in four samples. **(B)** Scatter plot of correlation between gene expression and mitochondrial gene expression ratio and gene number. **(C)** Spatial distribution of gene expression in four samples. **(D)** Spatial distribution of numbers of expressed genes in four samples.

Supplementary Figure 2
**(A)** Number of differentially expressed genes in all clusters. **(B)** Heatmap of top 10 differentially expressed genes in all clusters.

Supplementary Figure 3 The co-essential relationship between differentially expressed hypoxia genes and top100 genes in cluster 0. **(A)** Correlation heatmap of hypoxia genes in cluster 0. **(B)** The network of CytoHubba calculation in cluster 0. **(C)** Co-essentiality network plot of differentially expressed hypoxia genes and top100 genes in cluster 0. The fill color of the node shows the ranking of Mcode score. Cyana blue background indicates Mcode group. Red letter indicates marker genes. Red border indicates differentially expressed hypoxia gene.

Supplementary Figure 4 The co-essential relationship between differentially expressed hypoxia genes and top100 genes in cluster 1. **(A)** Correlation heatmap of hypoxia genes in cluster 1. **(B)** The network of CytoHubba calculation in cluster 1. **(C)** Co-essentiality network plot of differentially expressed hypoxia genes and top100 genes in cluster 1. The fill color of the node shows the ranking of Mcode score. Cyana blue background indicates Mcode group. Red letter indicates marker genes. Red border indicates differentially expressed hypoxia gene.

Supplementary Figure 5 The co-essential relationship between differentially expressed hypoxia genes and top100 genes in cluster 6. **(A)** Correlation heatmap of hypoxia genes in cluster 6. **(B)** The network of CytoHubba calculation in cluster 6. **(C)** Co-essentiality network plot of differentially expressed hypoxia genes and top100 genes in cluster 6. The fill color of the node shows the ranking of Mcode score. Cyana blue background indicates Mcode group. Red letter indicates marker genes. Red border indicates differentially expressed hypoxia gene.

Supplementary Figure 6 The co-essential relationship between differentially expressed hypoxia genes and top100 genes in cluster 5. **(A)** Correlation heatmap of hypoxia genes in cluster 5. **(B)** The network of CytoHubba calculation in cluster 5. **(C)** Co-essentiality network plot of differentially expressed hypoxia genes and top100 genes in cluster 5. The fill color of the node shows the ranking of Mcode score. Cyana blue background indicates Mcode group. Red letter indicates marker genes. Red border indicates differentially expressed hypoxia gene.

Supplementary Figure 7 The string map image of differentially expressed genes in cluster 10.

Supplementary Figure 8 The characteristics of KEGG enrichment pathway of cluster 0, cluster 1, cluster 6, cluster 5, and cluster 10.

Supplementary Figure 9 Multivariate Cox proportional hazards regression model for hypoxia-dependent spatial clusters score and clinicopathological factors in **(A)** basal-like, **(B)** HER2-enriched, **(C)** luminal A, **(D)** luminal B, and **(E)** normal-like subtypes.

Supplementary Figure 10 Comparison of infiltrated immune cells in different breast cancer subtypes of 1904 breast cancers from METABRIC database.

Supplementary Figure 11 The relationship between hypoxia-dependent spatial clusters scores, immune cells infiltration and immune function in human claudin-low breast cancer. **(A)** The correlation of hypoxia-dependent spatial clusters score of cluster 0, cluster 1, cluster 6, cluster 5, and cluster 10 with infiltrated immune cells score in claudin-low subtype. **(B)** The correlation of cluster 0, cluster 1, cluster 6, cluster 5, and cluster 10 score with immune function score in claudin-low subtype. **(C)** The comparation of infiltrated immune cells score of low and high groups of cluster 0 and cluster 6 gene score in claudin-low subtype. **(D)** The comparation of immune function score of low and high groups of cluster 0 and cluster 6 gene score in claudin-low subtype. * *p* < 0.05, ** *p* < 0.01, *** *p* < 0.005.

Supplementary Figure 12 Check point genes expression in hypoxia-dependent spatial clusters and their clinival relevance.**(A)**Expression-level scaled heatmap of check point genes of cluster 0, cluster 1, cluster 6, cluster 5, and cluster 10. **(B)** The comparation of CD276,CD40,CD274,CD44,and NRP1 expression of low and high groups of cluster 0 ssGSEA score in claudin-low subtype. **(C)** The comparation of CD274,CD40,CD276,CD44,and TNFSF9 expression of low and high groups of cluster 6 ssGSEA score in claudin-low subtype. **(D)** Kaplan-Meier overall survival plot of CD276, NRP1, and CD44 expression in claudin-low subtype. **(E)** Kaplan-Meier relapse-free survival plot of CD276, NRP1, and CD44 expression in claudin-low subtype. * *p* < 0.05, ** *p* < 0.01, *** *p* < 0.005.

Supplementary Figure 13 Hypoxia induces spatial distribution remodeling of immune microenvironment in claudin-low breast cancer. Hypoxia remodels the spatial heterogeneity. The tumor from center to periphery were enriched into five hypoxia-dependent subgroups with differentially expressed genes, which were matched to necrosis, necrosis periphery, hypoxic tumor, adaptive survival tumor, and invasive tumor, respectively. In the invasive tumor, hypoxia promotes CD276 and NPR1 expression. CD276 binds to CD276 receptor to inhibit CD8+ T lymphocytes infiltration. NRP1 overexpression accelerated CD8+ T cell exhaustion in invasive tumor. Lack of insufficient infiltration of activated CD8+ T cells into the tumor microenvironment leads to unresponsiveness to immunotherapy.
