# Supplementary figures and images for: The relevance between hypoxia-dependent spatial transcriptomics and the prognosis and efficacy of immunotherapy in claudin-low breast cancer
Source: Front Immunol. 2023 Jan 4;13:1042835. doi: 10.3389/fimmu.2022.1042835 (PMC9846556; doi:10.3389/fimmu.2022.1042835)

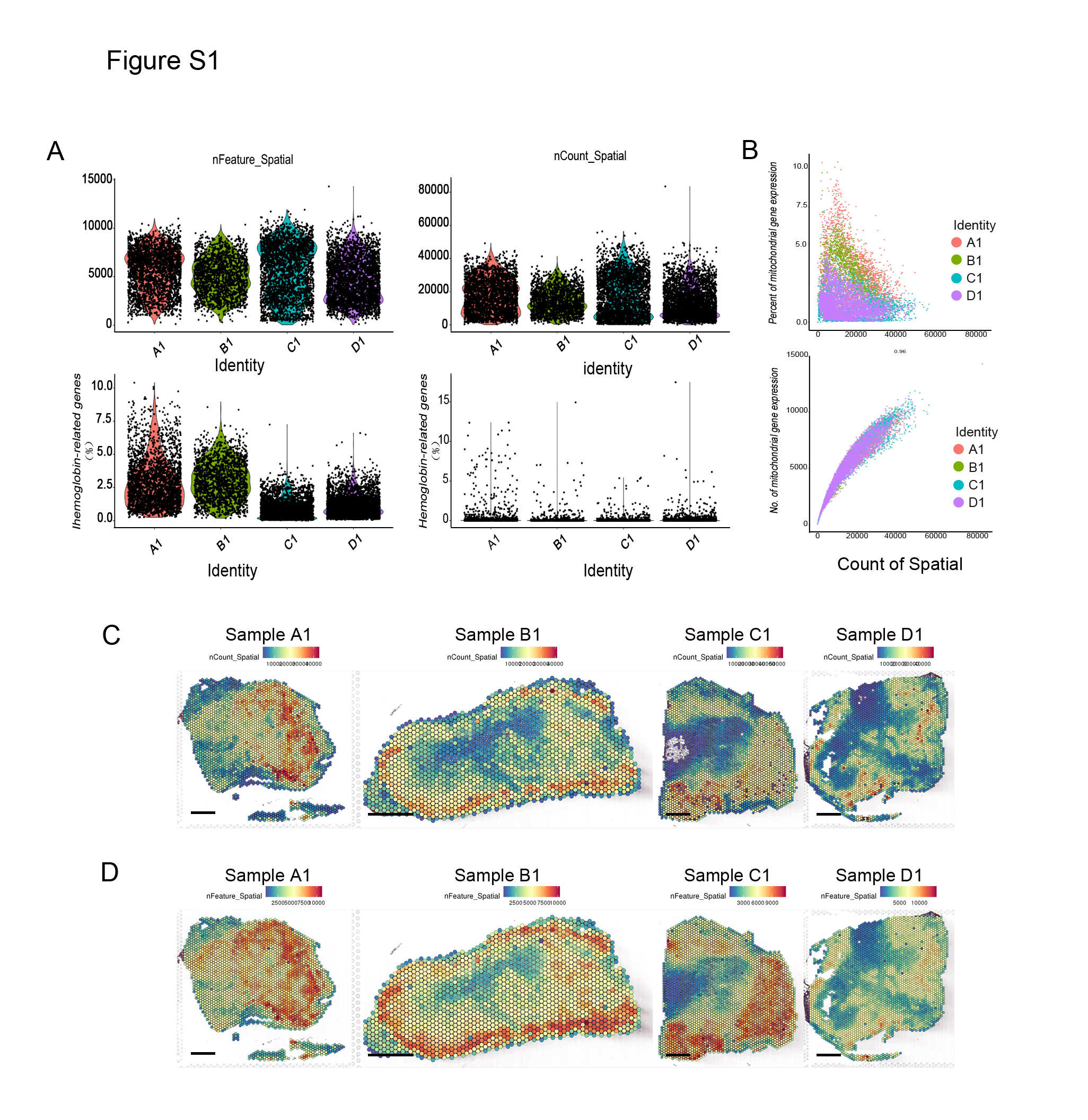

Supplement: Supplementary Figure 1 — A Spatial Transcriptomic Atlas of human claudin-low MDA-MB-231 engrafts. (A) Distribution of all expression gene numbers in four samples, distribution of all counts in four samples, distribution of mitochondrial gene in four samples, distribution of hemoglobin gene expression in four samples. (B) Scatter plot of correlation between gene expression and mitochondrial gene expression ratio and gene number. (C) Spatial distribution of gene expression in four samples. (D) Spatial distribution of numbers of expressed genes in four samples. [file Image_1.jpeg]

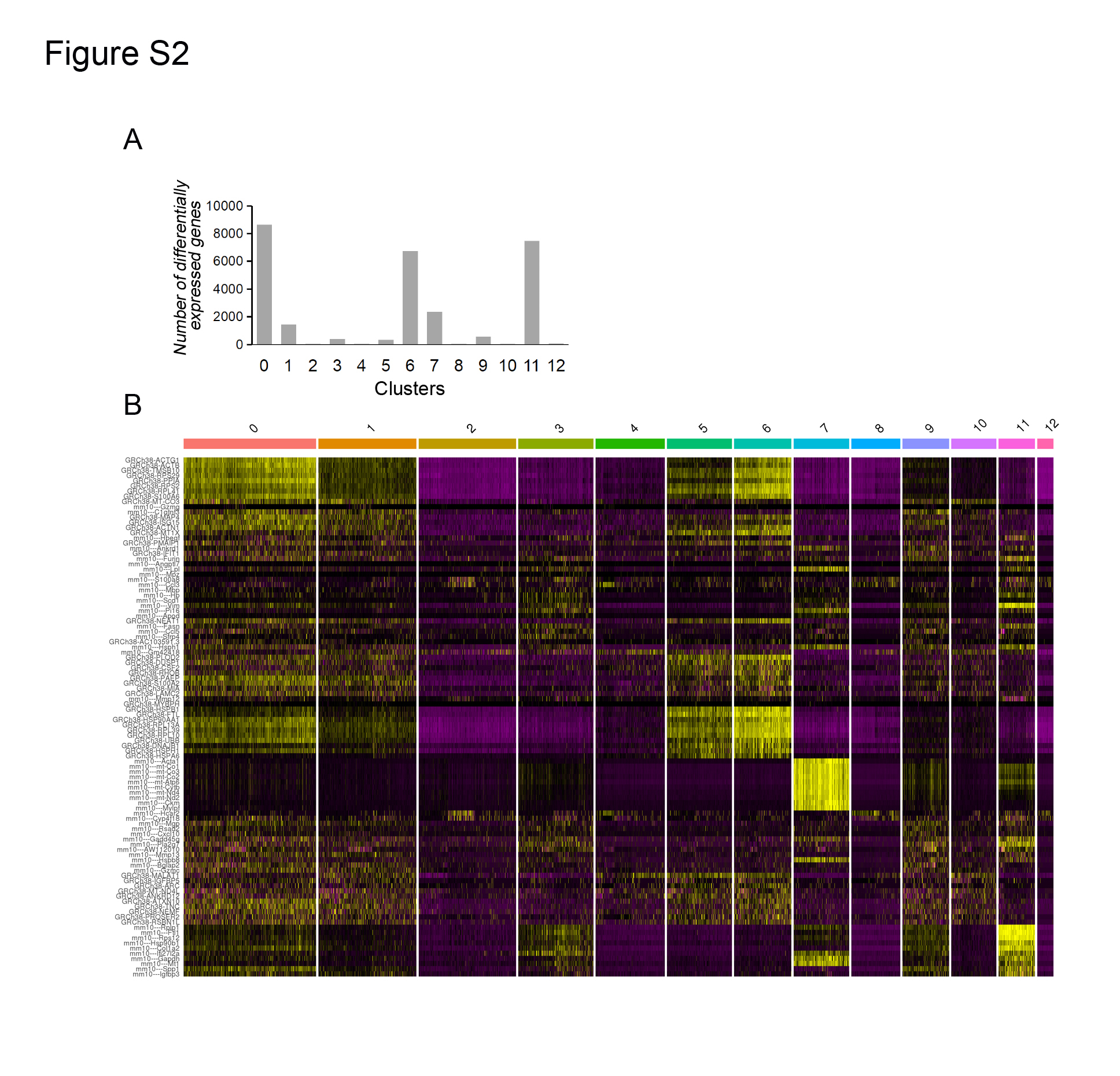

Supplement: Supplementary Figure 2 — (A) Number of differentially expressed genes in all clusters. (B) Heatmap of top 10 differentially expressed genes in all clusters. [file Image_2.jpeg]

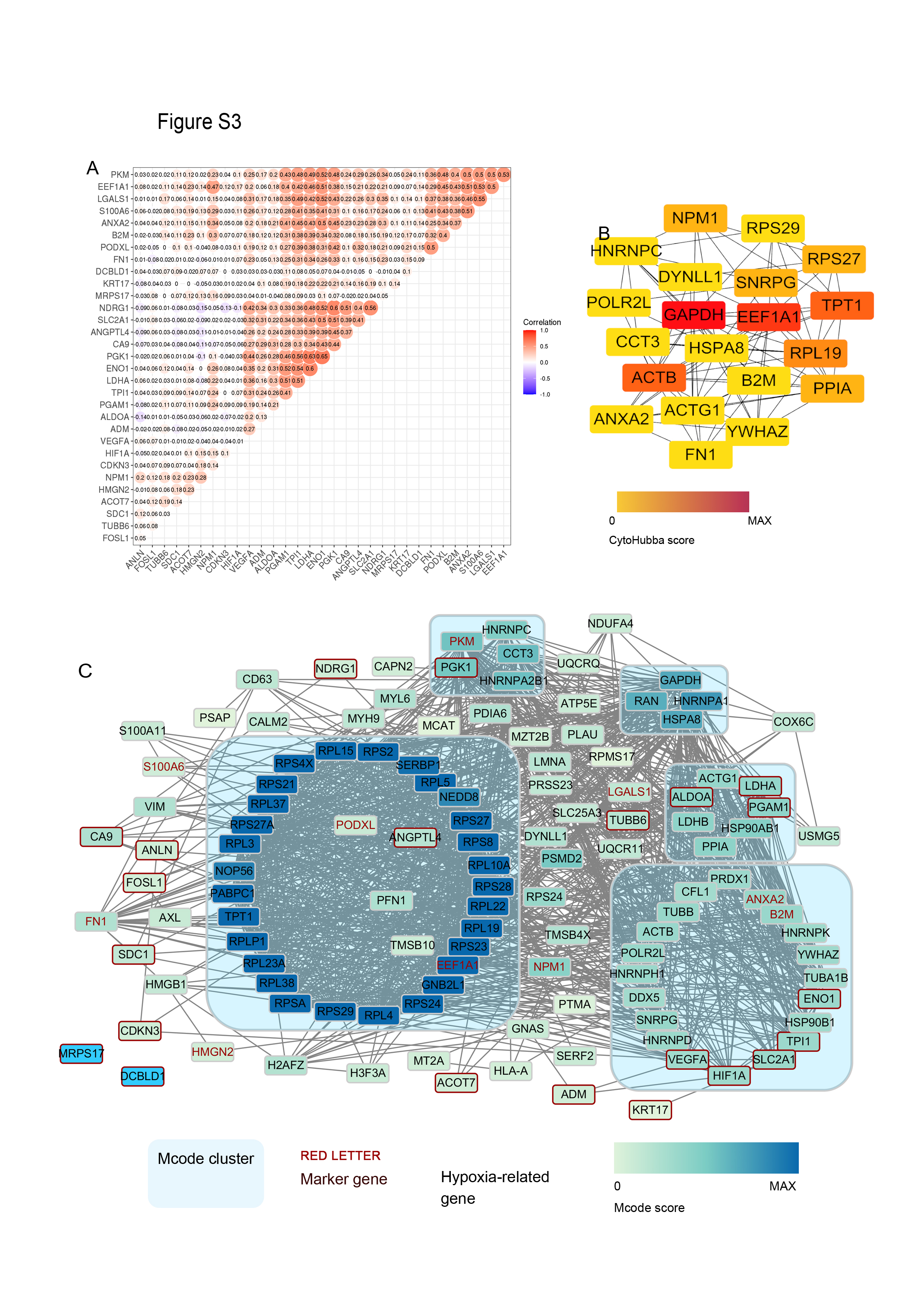

Supplement: Supplementary Figure 3 — The co-essential relationship between differentially expressed hypoxia genes and top100 genes in cluster 0. (A) Correlation heatmap of hypoxia genes in cluster 0. (B) The network of CytoHubba calculation in cluster 0. (C) Co-essentiality network plot of differentially expressed hypoxia genes and top100 genes in cluster 0. The fill color of the node shows the ranking of Mcode score. Cyana blue background indicates Mcode group. Red letter indicates marker genes. Red border indicates differentially expressed hypoxia gene. [file Image_3.jpeg]

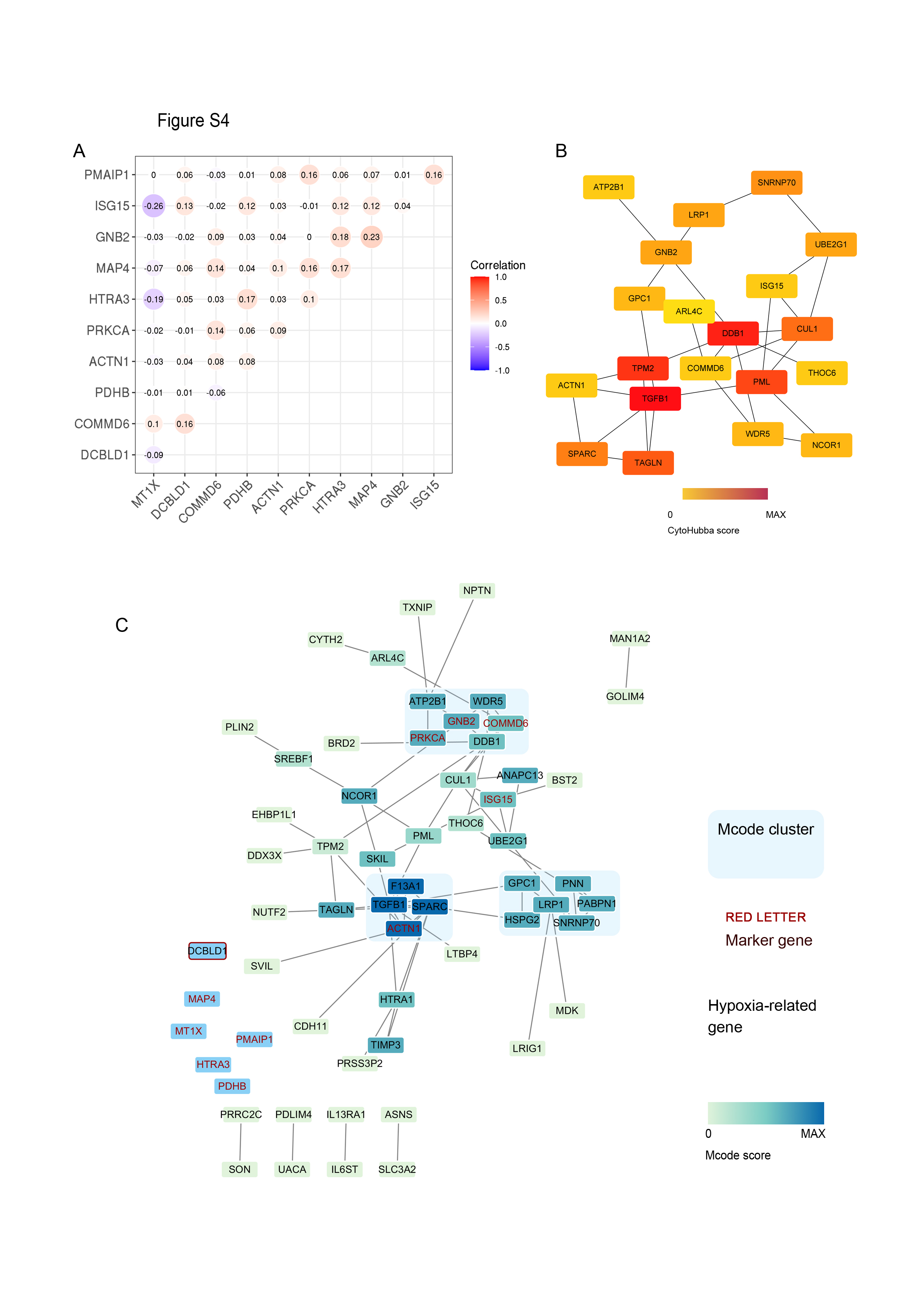

Supplement: Supplementary Figure 4 — The co-essential relationship between differentially expressed hypoxia genes and top100 genes in cluster 1. (A) Correlation heatmap of hypoxia genes in cluster 1. (B) The network of CytoHubba calculation in cluster 1. (C) Co-essentiality network plot of differentially expressed hypoxia genes and top100 genes in cluster 1. The fill color of the node shows the ranking of Mcode score. Cyana blue background indicates Mcode group. Red letter indicates marker genes. Red border indicates differentially expressed hypoxia gene. [file Image_4.jpeg]

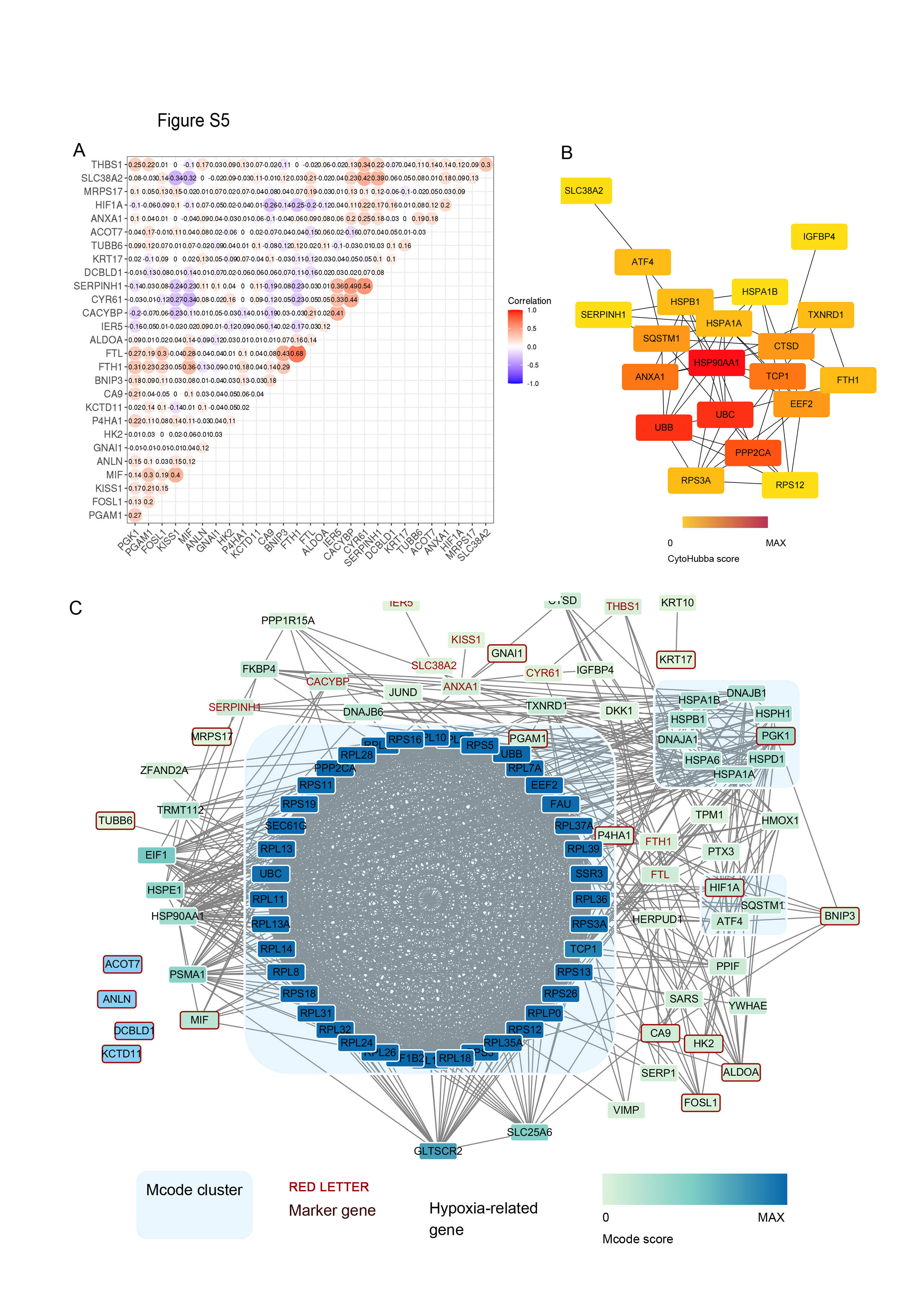

Supplement: Supplementary Figure 5 — The co-essential relationship between differentially expressed hypoxia genes and top100 genes in cluster 6. (A) Correlation heatmap of hypoxia genes in cluster 6. (B) The network of CytoHubba calculation in cluster 6. (C) Co-essentiality network plot of differentially expressed hypoxia genes and top100 genes in cluster 6. The fill color of the node shows the ranking of Mcode score. Cyana blue background indicates Mcode group. Red letter indicates marker genes. Red border indicates differentially expressed hypoxia gene. [file Image_5.jpeg]

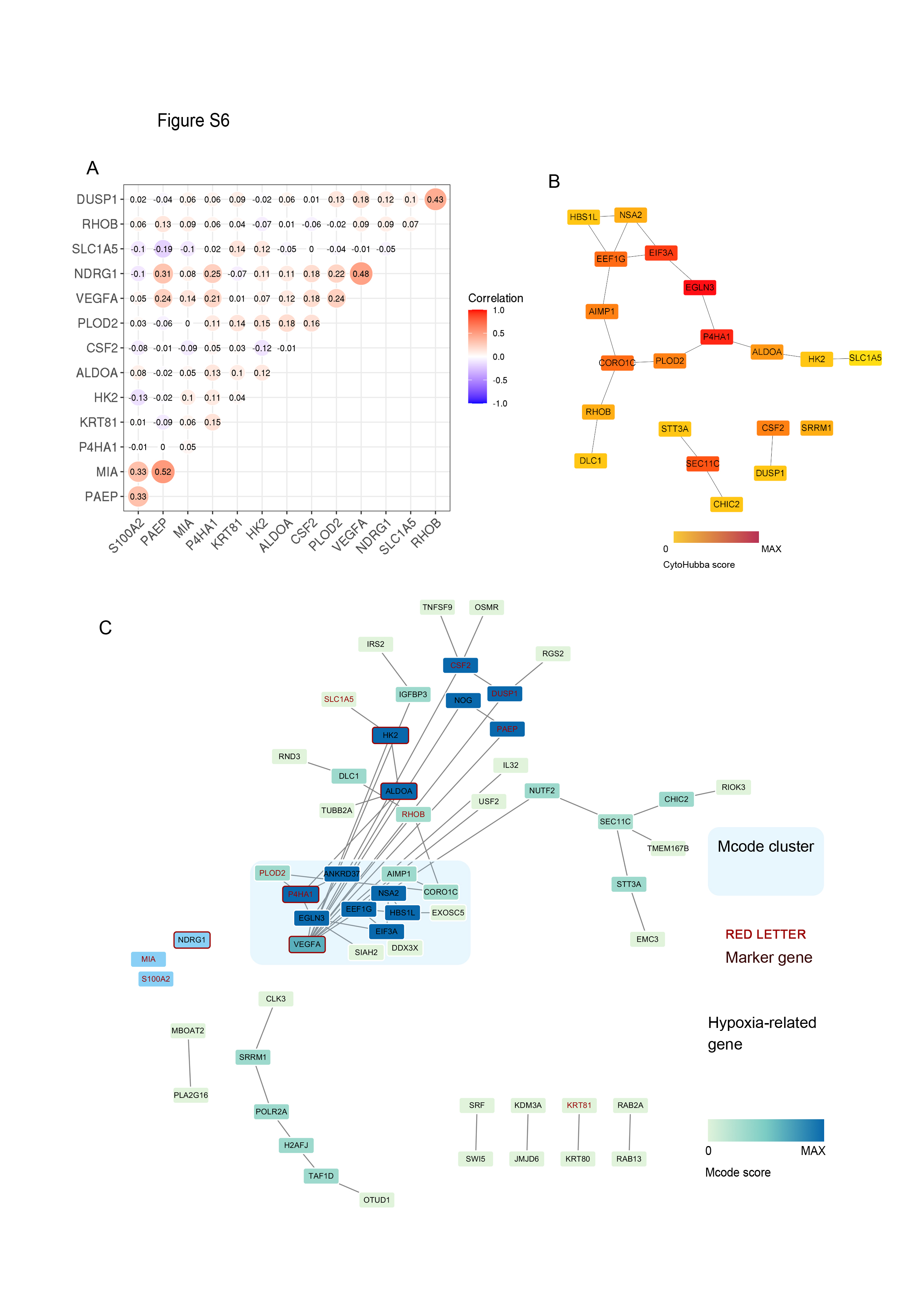

Supplement: Supplementary Figure 6 — The co-essential relationship between differentially expressed hypoxia genes and top100 genes in cluster 5. (A) Correlation heatmap of hypoxia genes in cluster 5. (B) The network of CytoHubba calculation in cluster 5. (C) Co-essentiality network plot of differentially expressed hypoxia genes and top100 genes in cluster 5. The fill color of the node shows the ranking of Mcode score. Cyana blue background indicates Mcode group. Red letter indicates marker genes. Red border indicates differentially expressed hypoxia gene. [file Image_6.jpeg]

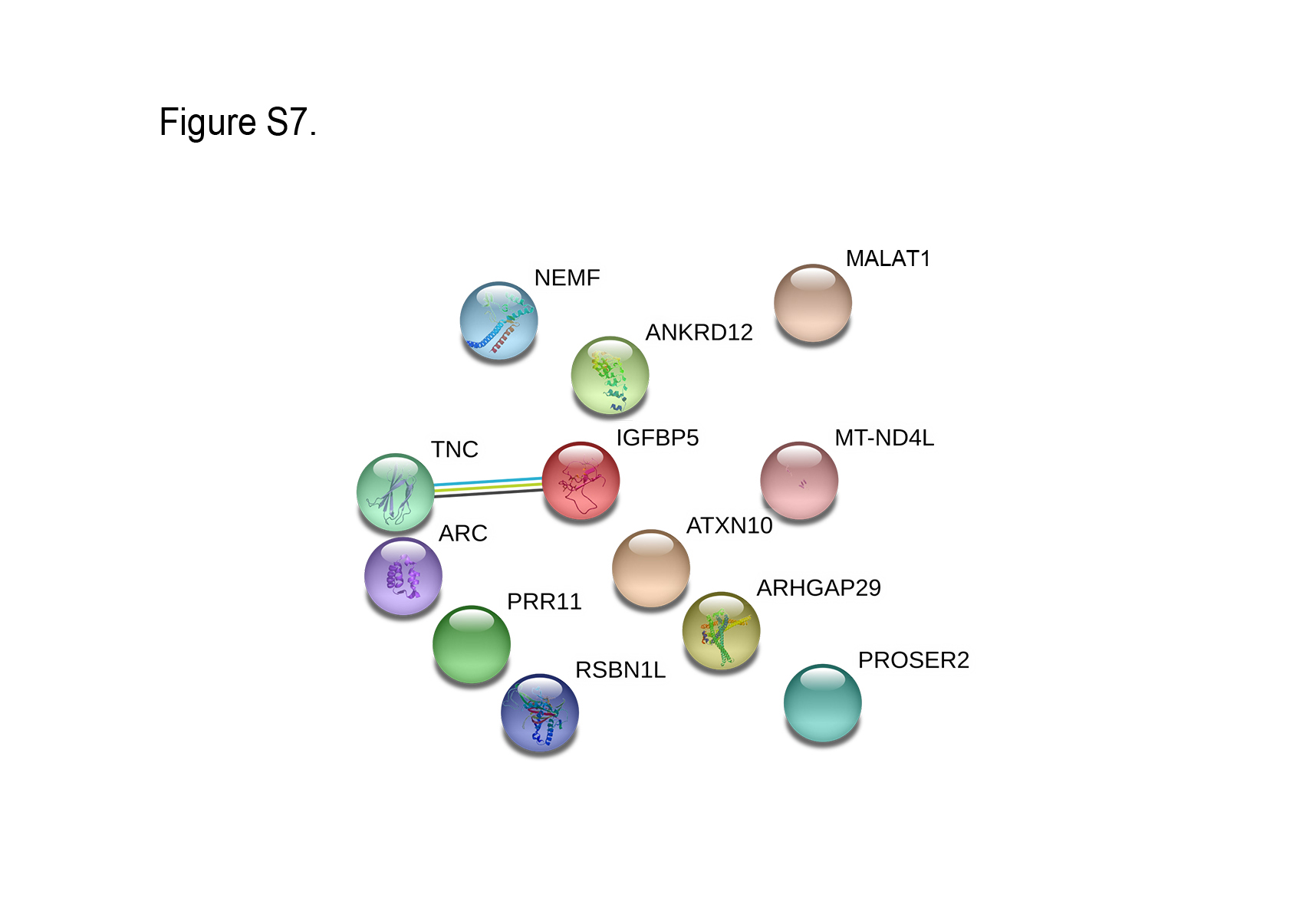

Supplement: Supplementary Figure 7 — The string map image of differentially expressed genes in cluster 10. [file Image_7.jpeg]

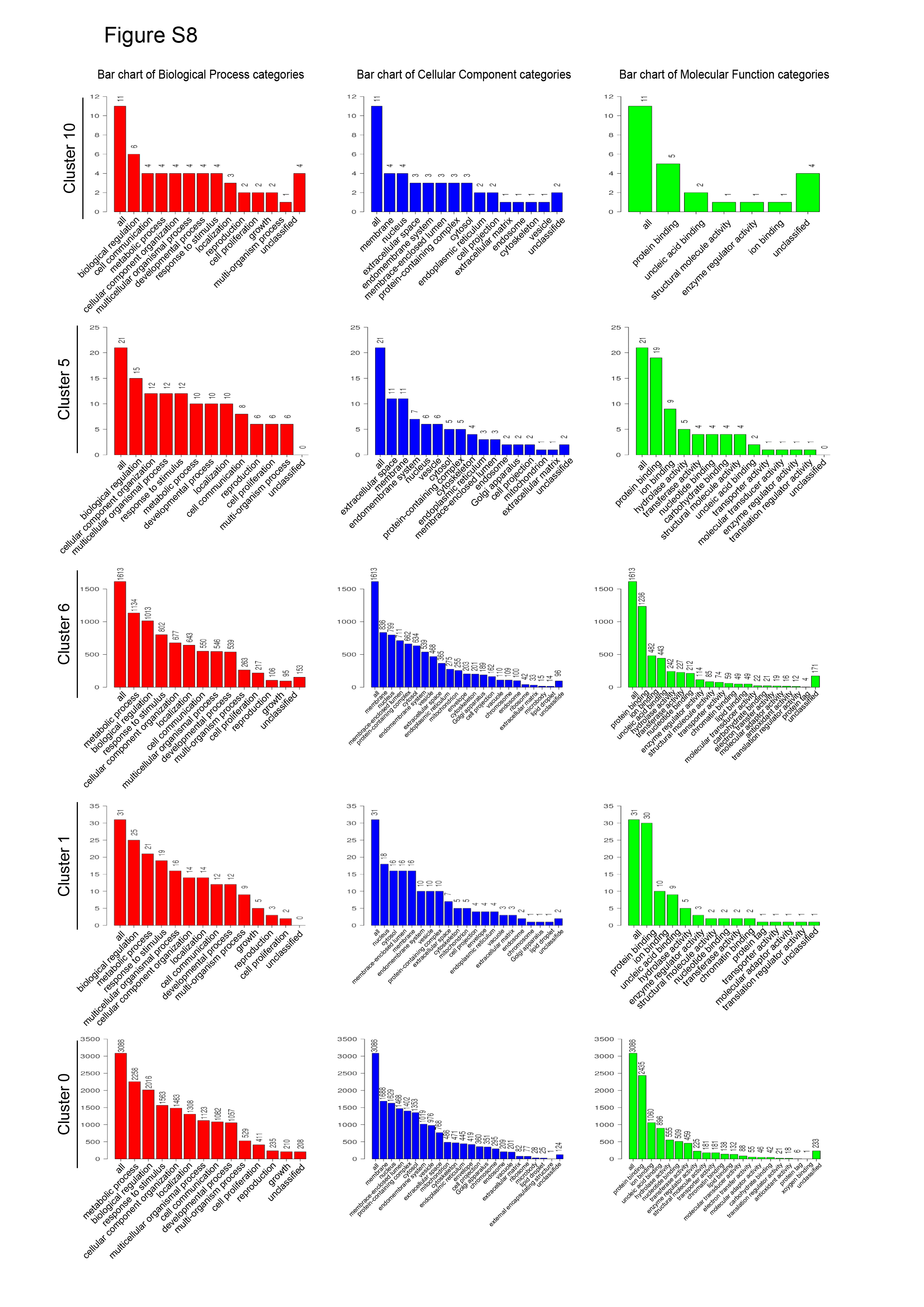

Supplement: Supplementary Figure 8 — The characteristics of KEGG enrichment pathway of cluster 0, cluster 1, cluster 6, cluster 5, and cluster 10. [file Image_8.jpeg]

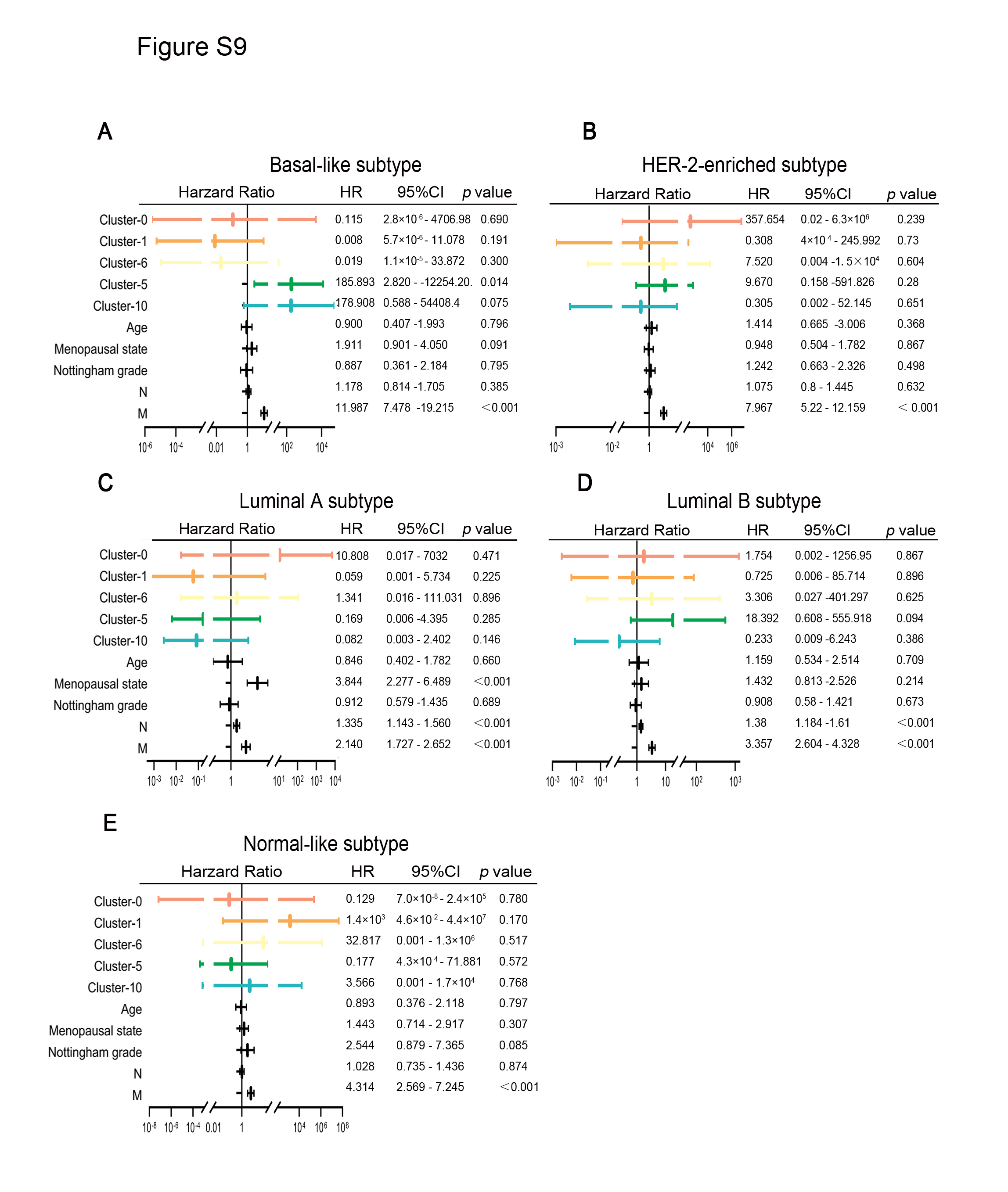

Supplement: Supplementary Figure 9 — Multivariate Cox proportional hazards regression model for hypoxia-dependent spatial clusters score and clinicopathological factors in (A) basal-like, (B) HER2-enriched, (C) luminal A, (D) luminal B, and (E) normal-like subtypes. [file Image_9.jpeg]

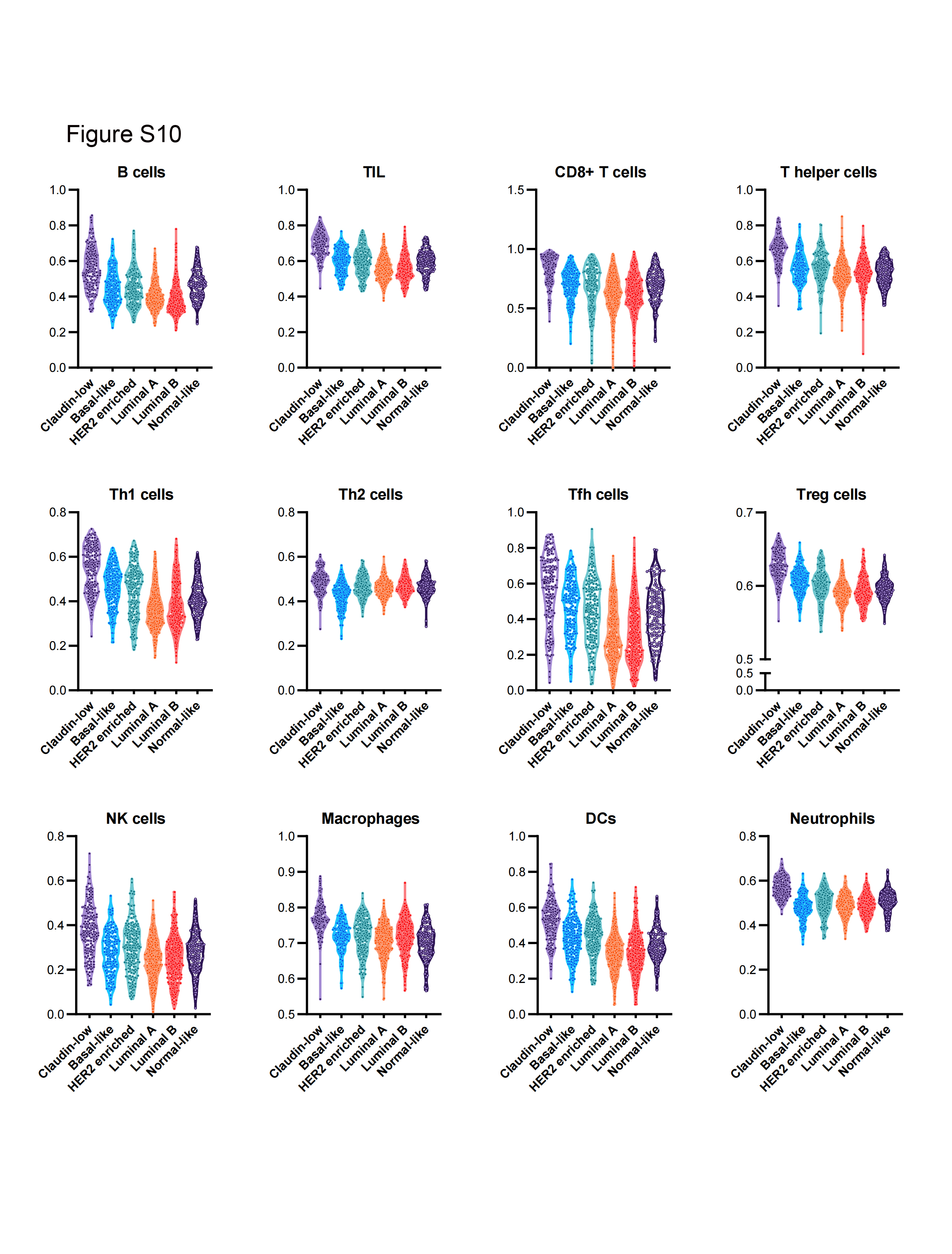

Supplement: Supplementary Figure 10 — Comparison of infiltrated immune cells in different breast cancer subtypes of 1904 breast cancers from METABRIC database. [file Image_10.jpeg]

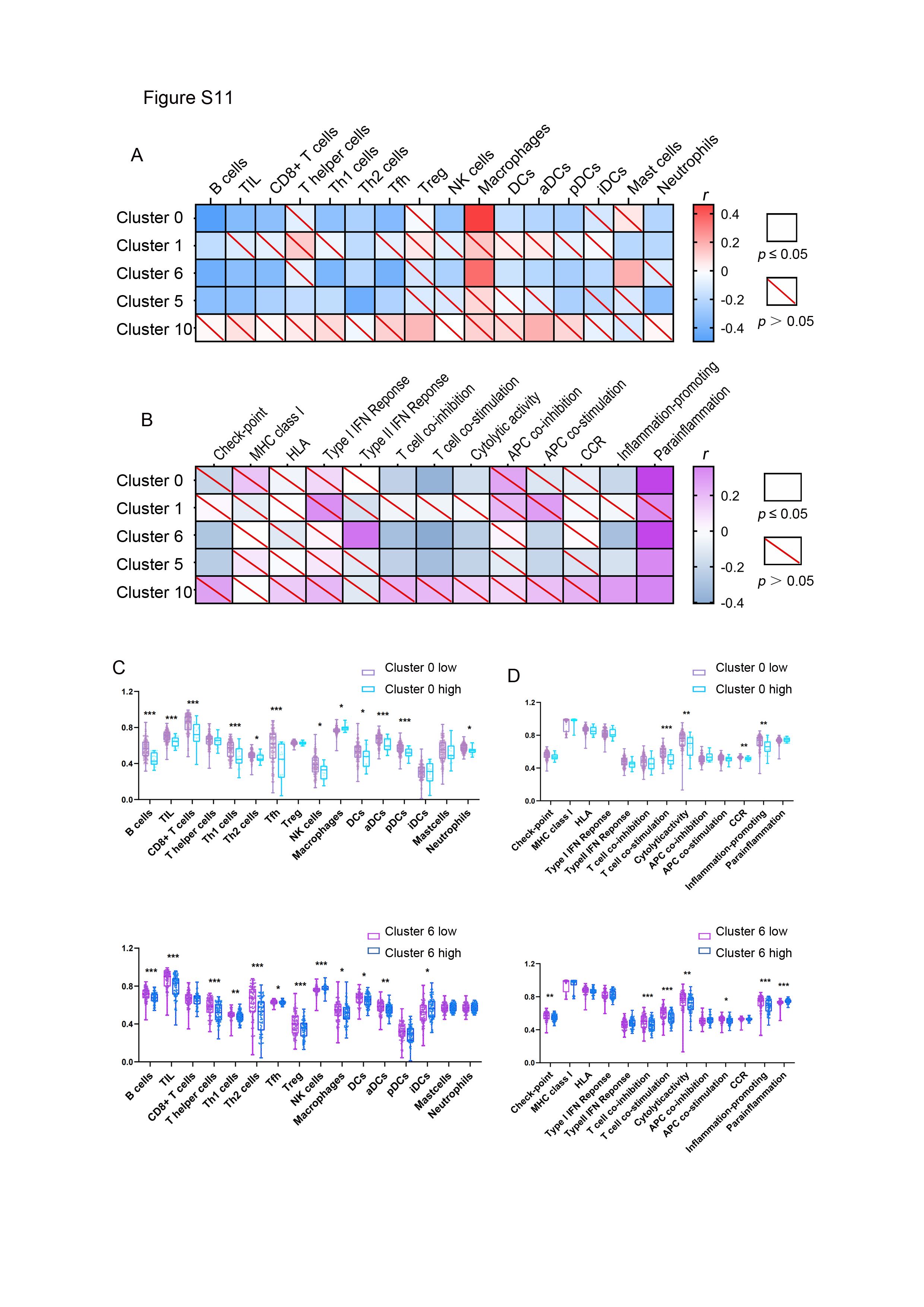

Supplement: Supplementary Figure 11 — The relationship between hypoxia-dependent spatial clusters scores, immune cells infiltration and immune function in human claudin-low breast cancer. (A) The correlation of hypoxia-dependent spatial clusters score of cluster 0, cluster 1, cluster 6, cluster 5, and cluster 10 with infiltrated immune cells score in claudin-low subtype. (B) The correlation of cluster 0, cluster 1, cluster 6, cluster 5, and cluster 10 score with immune function score in claudin-low subtype. (C) The comparation of infiltrated immune cells score of low and high groups of cluster 0 and cluster 6 gene score in claudin-low subtype. (D) The comparation of immune function score of low and high groups of cluster 0 and cluster 6 gene score in claudin-low subtype. * p < 0.05, ** p < 0.01, *** p < 0.005. [file Image_11.jpeg]

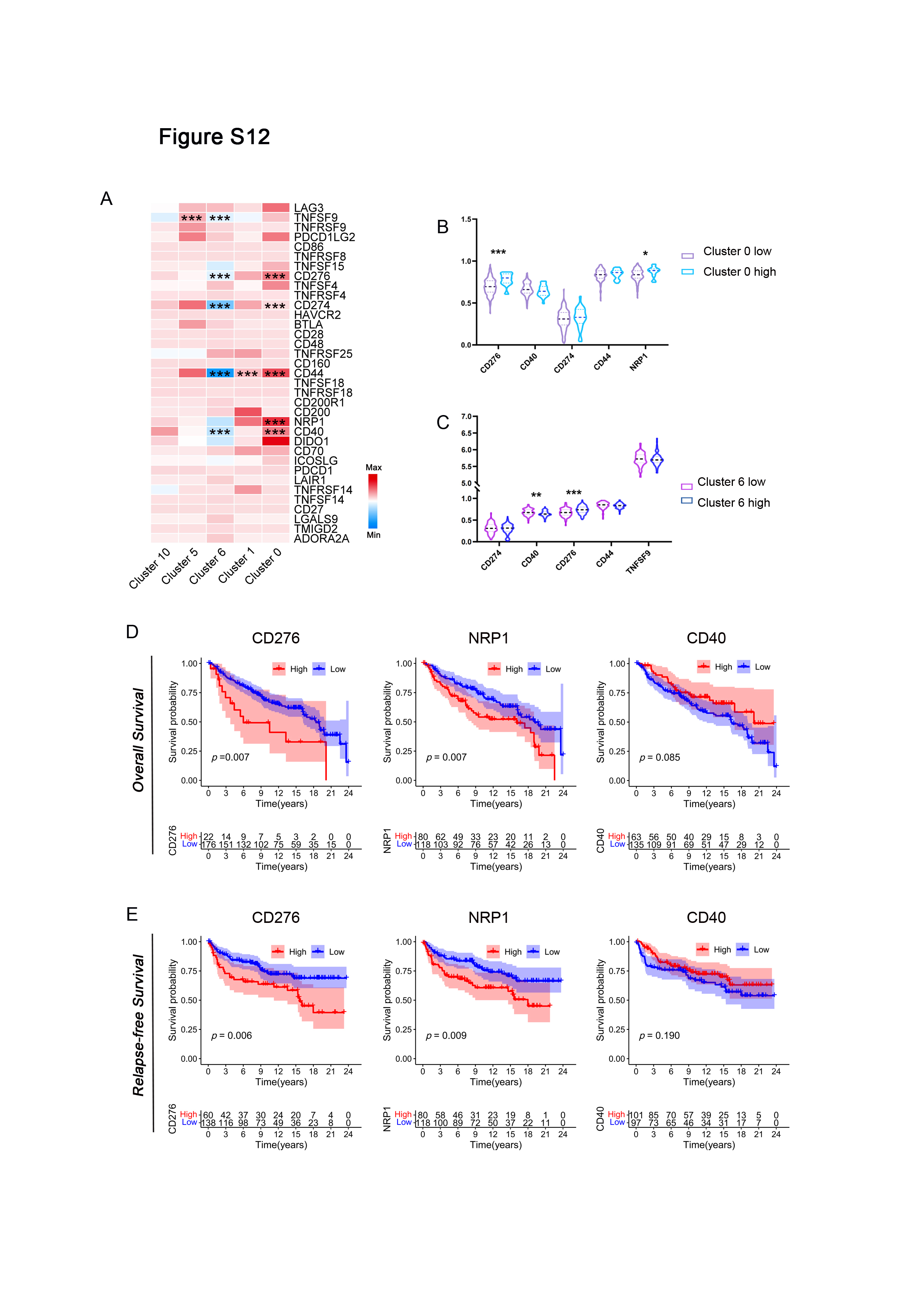

Supplement: Supplementary Figure 12 — Check point genes expression in hypoxia-dependent spatial clusters and their clinival relevance.(A)Expression-level scaled heatmap of check point genes of cluster 0, cluster 1, cluster 6, cluster 5, and cluster 10. (B) The comparation of CD276,CD40,CD274,CD44,and NRP1 expression of low and high groups of cluster 0 ssGSEA score in claudin-low subtype. (C) The comparation of CD274,CD40,CD276,CD44,and TNFSF9 expression of low and high groups of cluster 6 ssGSEA score in claudin-low subtype. (D) Kaplan-Meier overall survival plot of CD276, NRP1, and CD44 expression in claudin-low subtype. (E) Kaplan-Meier relapse-free survival plot of CD276, NRP1, and CD44 expression in claudin-low subtype. * p < 0.05, ** p < 0.01, *** p < 0.005. [file Image_12.jpeg]

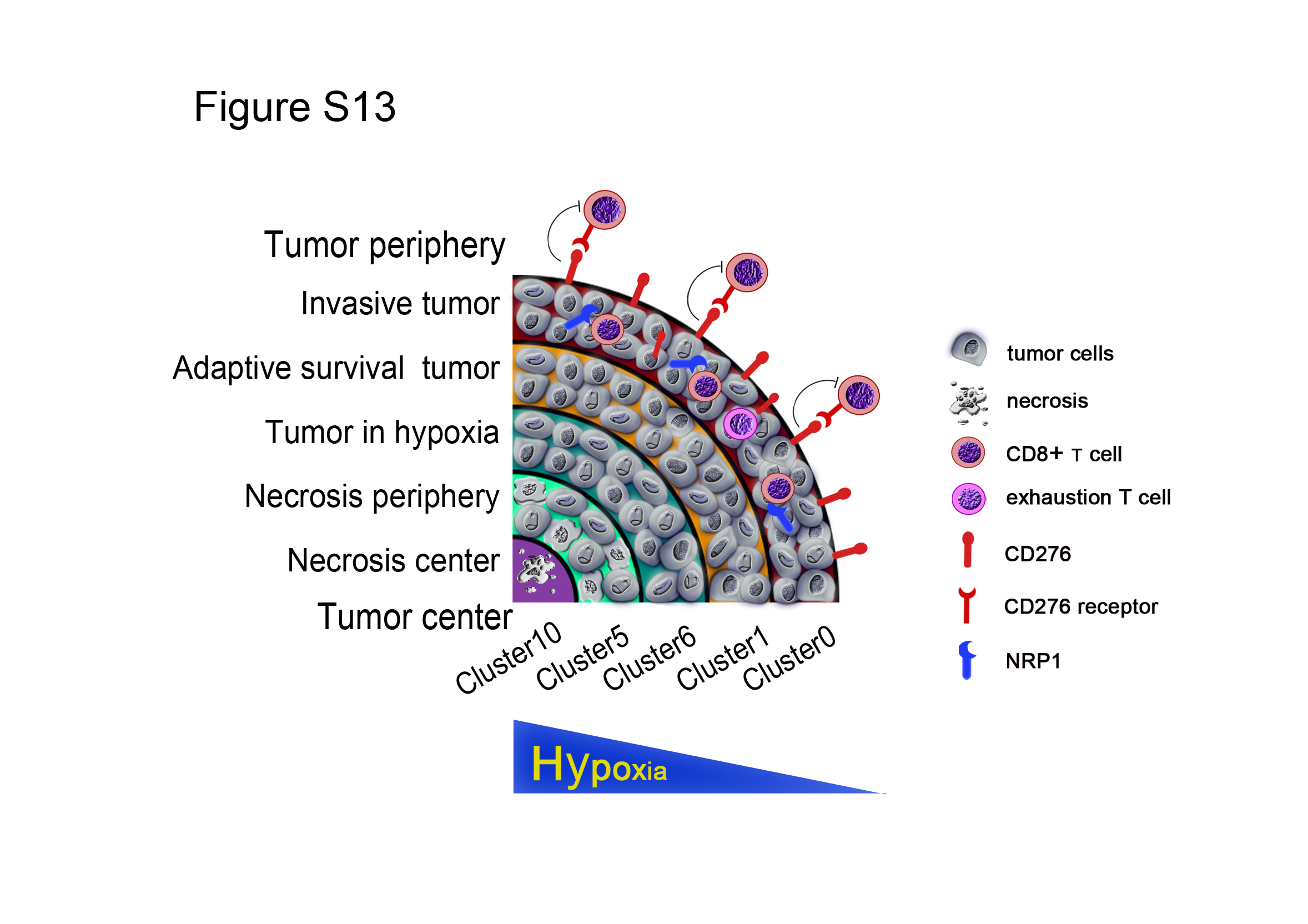

Supplement: Supplementary Figure 13 — Hypoxia induces spatial distribution remodeling of immune microenvironment in claudin-low breast cancer. Hypoxia remodels the spatial heterogeneity. The tumor from center to periphery were enriched into five hypoxia-dependent subgroups with differentially expressed genes, which were matched to necrosis, necrosis periphery, hypoxic tumor, adaptive survival tumor, and invasive tumor, respectively. In the invasive tumor, hypoxia promotes CD276 and NPR1 expression. CD276 binds to CD276 receptor to inhibit CD8+ T lymphocytes infiltration. NRP1 overexpression accelerated CD8+ T cell exhaustion in invasive tumor. Lack of insufficient infiltration of activated CD8+ T cells into the tumor microenvironment leads to unresponsiveness to immunotherapy. [file Image_13.jpeg]
