# Supplementary material for: The relevance between hypoxia-dependent spatial transcriptomics and the prognosis and efficacy of immunotherapy in claudin-low breast cancer
Source: Front Immunol. 2023 Jan 4;13:1042835. doi: 10.3389/fimmu.2022.1042835 (PMC9846556; doi:10.3389/fimmu.2022.1042835)
Supplement: Supplementary file 14 [file Table_1.doc]

Supplementary Table 1. Information of primary antibodies used in this study

| Antibody | Source | NO. | Company | Dilution |
| --- | --- | --- | --- | --- |
| LDHA | Rabbit | PA5-81292 | Invitrogen | 1:200 |
| P4HA1 | Rabbit | ab244302 | Abcam | 1:100 |
| BNIP3 | Rabbit | #44060S | CST | 1:300 |
| CA9 | Rabbit | #5649 | CST | 1:200 |
| Endomucin | Rat | 11-5851-80 | eBioscience | 1:400 |
