# Supplementary material for: The relevance between hypoxia-dependent spatial transcriptomics and the prognosis and efficacy of immunotherapy in claudin-low breast cancer
Source: Front Immunol. 2023 Jan 4;13:1042835. doi: 10.3389/fimmu.2022.1042835 (PMC9846556; doi:10.3389/fimmu.2022.1042835)
Supplement: Supplementary file 15 [file Table_2.docx]

Supplementary Table 2. Hypoxia-related genes analyzed in this study

| Hgnc symbol | Names | Function |
| --- | --- | --- |
| *VEGFA* | Vascular endothelial growth factor A | VEGF signalling |
| *SLC2A1* | Solute carrier family 2, member 1 | Adipocytokine signalling |
| *PGAM1* | Phosphoglycerate mutase 1 | Glucose metabolism |
| *ENO1* | Enolase 1 | Glucose metabolism |
| *LDHA* | Lactate dehydrogenase A | Glucose metabolism |
| *TPI1* | Triosephosphate isomerase 1 | Glucose metabolism |
| *P4HA1* | Prolyl 4-hydroxylase,α-polypeptide I | Extracellular matrix metabolism |
| *MRPS17* | Mitochondrial ribosomal protein S17 | Mitochondrial translation |
| *CDKN3* | Cyclin-dependent kinase inhibitor 3 | Cellular proliferation |
| *ADM* | Adrenomedullin | Signal transduction |
| *NDRG1* | N-myc downstream regulated 1 | Response to metalion |
| *TUBB6* | Tubulin, β6 | Gap junction |
| *ALDOA* | Aldolase A, fructose-bisphosphate | Glucose metabolism |
| *MIF* | Macrophage migration inhibitory factor | Tyrosine metabolism |
| *ACOT7* | Acyl-CoA thioesterase 7 | Lipid metabolism |
| *BNIP3* | BCL2/adenovirus E1B 19 kDa protein-interacting protein 3 | pro-apoptosis |
| *CA9* | carbonic anhydrase 9 | pH regulation |
| *PGK1* | phosphoglycerate kinase 1 | Glucose metabolism |
| *HK2* | Hexokinase2 | Glycolysis, gluconeogenesis, energy pathway |
| *ANGPTL4* | angiopoietin-like 4 | Lipid and glucose metabolism |
| *ANLN* | anillin | Cytokinesis |
| *BNC1* | basonuclin 1 | Keratinocyte proliferation |
| *C20orf20* | chromosome 20 open reading frame 20 | Cellular proliferation |
| *COL4A6* | collagen, type IV, alpha 6 | Extracellular matrix metabolism |
| *DCBLD1* | discoidin,CUB and LCCL domain containing 1 | Unknown |
| *FAM83B* | family with sequence similarity 83, member B | EGFR signaling pathway; RAS/MAPK,PI3K/AKT/TOR downstream signaling pathway |
| *FOSL1* | FOS-like antigen 1 | Cellular proliferation |
| *GNAI1* | guanine nucleotide binding protein | Signal transduction |
| *HIG2* | hypoxia-inducible gene 2 | Stress response |
| *KCTD11* | potassium channel tetramerization domain containing 11 | Apoptosis |
| *KRT17* | keratin17 | Keratin production |
| *SDC1* | syndecan 1 | Cellular proliferation |
| *SLC16A1* | solute carrier family 16 member 1 (monocarboxylic acid ransporter 1) | Glucose metabolism |
| *HIF1A* | hypoxia-inducible factor 1 alpha | Transciptional regulator of the adaptive response to hypoxia |
| *HIF2* | EPAS1;Endothelial PAS Domain Pritein 1 | Regulating VEGF expression |
