# Supplementary material for: The relevance between hypoxia-dependent spatial transcriptomics and the prognosis and efficacy of immunotherapy in claudin-low breast cancer
Source: Front Immunol. 2023 Jan 4;13:1042835. doi: 10.3389/fimmu.2022.1042835 (PMC9846556; doi:10.3389/fimmu.2022.1042835)
Supplement: Supplementary file 19 [file DataSheet_1.docx]

**Hypoxia-dependent spatial transcriptomics predicted the prognosis and efficacy of immunotherapy in claudin-low breast cancer**

Huizhi Sun^1,2^, Yanlei Li^1^, Yanhui Zhang^2^_,_ Xiulan Zhao^1^, Xueyi Dong^1^, Yuhong Guo^2^_,_ Jing Mo^1^, Na Che^1^, Xinchao Ban^1^, Fan Li^1^, Xiaoyu Bai^1^, Yue Li^1^,Danfang Zhang^1^*, Jihui Hao^2^*

1 Tianjin Medical University Cancer Institute and Hospital, National Clinical Research Center for Cancer, Key Laboratory of Cancer Prevention and Therapy, Tianjin’s Clinical Research Center for Cancer, Tianjin 300060, China

2 Department of Pathology, Tianjin Medical University, Tianjin 300070, China

Running title: *ST feature of Hypoxia-induced heterogeneity in claudin-low breast cancer*

* Corresponding author: **Danfang Zhang**, Department of Pathology, Tianjin Medical University and General Hospital, Tianjin 300070, PR China, [Tel:+86 2283336813](D:/Documents/iResearch%20at%20TMU/Projects%20ing/2020%20visium%20spatial/Results/Figures%202022-7-13/Tel:+8613821015919), E-mail: [zhangdf@tmu.edu.cn](mailto:zhangdf@tmu.edu.cn); **Jihui Hao**, Department of Pancreatic Cancer, Tianjin Medical University Cancer Institute and Hospital, Tianjin 300060, China. Tel:+86 22 23340123; E-mail: [haojihui@tjmuch.com](mailto:haojihui@tjmuch.com)

**Supplementary materials and methods**

**Immunohistochemical staining (IHC)**

Paraffin-embedded sections were stained immunohistochemically. The sections were deparaffinized in xylene and rehydrated through graded alcohols into water. Endogenous peroxidase was blocked with 3% hydrogen peroxide in 50% methanol for 10 min at room temperature. After rehydrating, the sections were washed with PBS and then pretreated with citrate buffer (0.01 M citric acid, pH 6.0) for 20 min at 100 ℃. After rinsing with PBS, slides were incubated with primary polyclonal antibodies, including the antibody against overnight at 4 ℃. Visualization was performed using diaminobenzidine (DAB). Appropriate positive and negative controls were included.

**Immunofluorescent staining**

Serial frozen sections were used for Immunofluorescent staining. Endogenous peroxidase was blocked using 5% goat serum at room temperature for 20 min. The slides were incubated with primary anti-endomucin and anti-pimonidazole antibodies over night at 4°C respectively, washed with PBS. An Alexa Fluor 680-labeled goat anti-rabbit secondary antibody (1:200, Invitrogen) and a Texas red-labeled goat anti-rat secondary antibody (1:200, Invitrogen) were incubated with tissues at room temperature for 2 h individually, followed by washing with PBS twice. Stained tissue sections were mounted with a Vectashield mounting medium (ZLI.9557, Zhongshan) and were analyzed by confocal microscopy (Nikon A1 Confocal microscope, Nikon).

**Spatial transcriptomics**

***Slide preparation***

Spatial Transcriptomics slides were printed with four capture areas (6.5 x 6.5 mm), each with 4999 capture spots of barcoded primers (10x Genomics). The spots have a diameter of 100 μm and are arranged in a centered rectangular lattice pattern. Every spot contains millions of oligonucleotides with the following features: a 30 nucleotide poly(dT) sequence for the capture of polyadenylated mRNA molecules; a 12 nucleotide unique molecular identifier (UMI) for the identification of duplicate molecules that arise during the library preparation and sequencing process; a 16 nucleotide Spatial Barcode, which is shared by all oligonucleotides within each individual gene expression spot; and a partial TruSeq Read 1 sequence, for use during the library preparation and sequencing mportions of the workflow.

***Tissue preparation***

The ST protocol was optimized for MDA-MB-23 engraft tissue according to 10x genomics. Four tumors were randomly selected for ST. Briefly, a quarter of each tumor was cut into 5mm-thickness tissue block and frozen in dry ice immediately. Tumor block embedded with OCT were cryosectioned at 10 μm thickness and attached on the capture areas to proceed to the next step.

***Fixation, staining and imaging***

Sectioned slides were incubated at 37℃ for 1 min, fixed in methanol for 10 min at -20℃. For staining, sections were incubated in isopropanol (Millipore Sigma) for 6 min, Mayer’s hematoxylin (Dako, Agilent, Santa Clara, CA) for 7 min, bluing buffer (Dako) for 1min, and Eosin (Sigma-Aldrich) diluted 1:5 in Tris-base (0.45M Tris, 0.5M acetic acid, pH 6.0) for 1 min. The slide was washed in deionized water after each of the staining steps. After air-drying, the slides were mounted with 85% glycerol and coverslip. H&E images were taken at 40× magnification using Digital slice scanner (Hamamatsu). The coverslip was removed after imaging by immersing slides in RNase and DNase free water.

***Tissue permeabilization***

The slides were inserted into slide cassettes to separate the tissue sections into individual reaction chambers (hereinafter wells). For pre-permeabilization, sections were incubated at 37℃ for 24 min with 70 μl permeabilization Enzyme. Wells were washed with 0.1 × SSC (Sigma-Aldrich).

***Reverse transcription, spatial library preparation and sequencing***

SSC was removed and 75 µl reverse transcription Master Mix was added to each well. Reverse transcription was conducted as ST recommended protocol. After RT, wells were washed with 0.1 × SSC. Sections were incubated in 75 µl 0.08 M KOH for 5 min at room temperature, and then were incubated in 75 µl Second Strand Mix for 15 min at 65℃. After removement of Second Strand Mix, 100 µl Buffer EB were added, and sections were in 35 µl 0.08 M KOH for 10 min at room temperature. The samples were transferred from each well to a corresponding tube containing Tris-HCl (1 M, pH 7.0). Next, 1 µl sample were added to the qPCR plate well containing the KAPA SYBR FAST qPCR Master Mix (KAPA Biosystems). A qPCR system was performed following protocol, and determine the optimal number of cycles. After that, 65 µl cDNA Amplification Mix were added into remaining sample. They were incubated with the recommended protocol for 12 cycles.

***Library preparation and RNA sequencing***

After the cDNA amplification products were qualified, the sequencing library was constructed with Library Construction KIT (10x Genomics). First, the cDNA was chemically knocked out. The cDNA fragment was cut into 200 ~ 300 BP fragments, and the cDNA fragment was segmented, terminal repair and an addition. The cDNA fragment was screened. The P7 adapter was connected and introduced into the sample index by PCR amplification. Finally, the sequence library was obtained. Sequencing was performed on Illumina Hiseq 3000/4000 with a 150bp pair-end run by Quick Biology (Pasadena, Ca). A data quality check was done on Illumina SAV. Demultiplexing was performed with Illumina Bcl2fastq2 v 2.17 program.

***RNA sequencing analysis***

In this study, 10x genomics official software Space Ranger 1.0.0 was used for data preprocessing, gene expression quantitative and point identification. Sequencing data preprocessing includes filtering the sequenced sequences, evaluating the quality of sequencing data, and calculating the sequence length distribution. Web-based ST Spot Detector Software Space ranger was used to identify the spatial barcode markers in Reads1 and UMI markers of different transcripts. Read2 were aligned to the reference genome, human GRCh38 v86 genome and mouse mm10 genome, using the transcriptome specific alignment software STAR, and selected the sequence with unique alignment position for subsequent analysis. Space Ranger obtains the bright field slide image of a single capture area and the fastq sequence, distinguished tissue and background, detected spot barcode. The gene spot matrix was generated by using visium spatial barcodes, and then point clustering and gene expression analysis were performed.

Software Seurat was used to analyze and cluster the four samples. The data with low quality were filtered. Principal component analysis (PCA), Uniform Manifold Approximation and Projection (UMAP) algorithm, were used to reduce the dimension of data and visualize data. All spots from four samples were clustered according to differentially expressed genes, which were expressed in over 25% spots with a value of LOGFC greater than 0.25. Heatmaps were generated with Seurat with default hierarchical clustering on read counts.

**Evaluation of hypoxia-dependent spatial clusters score**

The breast cancer hypoxia-dependent spatial clusters score was calculated based on the single-sample gene-set enrichment analysis (ssGSEA) using the marker gene set to quantify the expression levels of these genes for 1904 human breast cancers（Supplementary table 5）（1, 2）. The immune signatures score was evaluated using the gene sets that represented different immune cells and immune signatures from several publications including HLA, TILs, immune cytolytic activity (CYT), and interferon (IFN) response（Supplementary table 5）(3-5). We estimated the breast cancer hypoxia-dependent spatial clusters score between normal-like, luminal A, luminal B, HER2-enriched, claudin-low and basal-like subtype in the METABRIC breast cancers.

**Statistical analysis**

We compared the overall survival (OS) and recurrence-free survival (RFS) of cancer patients separated by the auto-selected hypoxia-dependent spatial clusters score in claudin-low breast cancer. Kaplan–Meier (K-M) analysis were carried out to compare the survival time differences using R language. *P*-values from log-rank tests were performed, and less than 0.05 was considered statistically significant. SPSS 20.0 software was used to build a Cox proportional hazards regression model of patients' age, tumor size, lymph node metastasis, distant metastasis and hypoxia-dependent spatial clusters score on the prognosis of breast cancer patients with different molecular subtype. The results were indicated in forest plots.

To compare the difference of hypoxia-dependent spatial clusters score in each breast cancer subtype, ANOVA test was carried out using SPSS 20.0 software. Chi-squared test was used to evaluate the difference of clinicopathological factors in the high and low score of cluster 0 marker gene in human claudin-low subtype. *Pearson* correlation was used to reveal the relationship between cluster-specific hypoxia genes and marker genes. The correlation heatmaps were provided by HIPLOT website (https://hiplot.com.cn/basic/cor-heatmap). The threshold of *P* < 0.05 was set up.

1. Curtis C, Shah SP, Chin SF, Turashvili G, Rueda OM, Dunning MJ, et al. The genomic and transcriptomic architecture of 2,000 breast tumours reveals novel subgroups. Nature. 2012;486(7403):346-52.

2．Pereira B, Chin SF, Rueda OM, Vollan HK, Provenzano E, Bardwell HA, et al. The somatic mutation profiles of 2,433 breast cancers refines their genomic and transcriptomic landscapes. Nat Commun. 2016;7:11479.

3. Mingyi Ju, Jia Bi, Qian Wei, Longyang Jiang, Qiutong Guan, Ming Zhang, et al. Pan-cancer analysis of NLRP3 inflammasome with potential implications in prognosis and immunotherapy in human cancer. Brief Bioinform. 2021;22:bbaa345.

4. Liu Z, Li M, Jiang Z, Wang X. A Comprehensive Immunologic Portrait of Triple-Negative Breast Cancer. Transl Oncol. 2018;11:311-329.

5. Lei T, Shen M, Deng X, Shi Y, Peng Y, Wang H, et al. Genomic characteristics of two breast malignant phyllodes tumors during pregnancy and lactation identified through whole-exome sequencing.

Orphanet J Rare Dis. 2022;17:382.

6. Yoshihara K, Shahmoradgoli M, Martínez E, Vegesna R, Kim H, Torres-Garcia W, et al. Inferring tumour purity and stromal and immune cell admixture from expression data. Nat Commun. 2013;4:2612.
